# Somatic variants for seed and fruit set in grapevine

**DOI:** 10.1186/s12870-021-02865-2

**Published:** 2021-03-13

**Authors:** Laura Costantini, Paula Moreno-Sanz, Chinedu Charles Nwafor, Silvia Lorenzi, Annarita Marrano, Fabiana Cristofolini, Elena Gottardini, Stefano Raimondi, Paola Ruffa, Ivana Gribaudo, Anna Schneider, Maria Stella Grando

**Affiliations:** 1 Research and Innovation Centre, Fondazione Edmund Mach, Via E. Mach 1, 38010 San Michele all’Adige, Italy; 2 Center Agriculture Food Environment (C3A), University of Trento, Via. E. Mach 1, 38010 San Michele all’Adige, Italy; 3 Center for Plant Science Innovation & Department of Biochemistry, University of Nebraska-Lincoln, Lincoln, NE USA; 4 Department of Plant Sciences, University of California, Davis, CA 95616 USA; 5 Institute for Sustainable Plant Protection - Research Council of Italy, Largo P. Braccini 2, 10095 Grugliasco, Italy

**Keywords:** *Vitis vinifera*, Seedlessness, Somatic variation, Reproductive development, Flower, Berry, Fertilization, Parthenocarpy, Stenospermocarpy, Single-nucleotide polymorphism

## Abstract

**Background:**

Grapevine reproductive development has direct implications on yield. It also impacts on berry and wine quality by affecting traits like seedlessness, berry and bunch size, cluster compactness and berry skin to pulp ratio. Seasonal fluctuations in yield, fruit composition and wine attributes, which are largely driven by climatic factors, are major challenges for worldwide table grape and wine industry. Accordingly, a better understanding of reproductive processes such as gamete development, fertilization, seed and fruit set is of paramount relevance for managing yield and quality. With the aim of providing new insights into this field, we searched for clones with contrasting seed content in two germplasm collections.

**Results:**

We identified eight variant pairs that seemingly differ only in seed-related characteristics while showing identical genotype when tested with the GrapeReSeq_Illumina_20K_SNP_chip and several microsatellites. We performed multi-year observations on seed and fruit set deriving from different pollination treatments, with special emphasis on the pair composed by Sangiovese and its seedless variant locally named Corinto Nero. The pollen of Corinto Nero failed to germinate in vitro and gave poor berry set when used to pollinate other varieties. Most berries from both open- and cross-pollinated Corinto Nero inflorescences did not contain seeds. The genetic analysis of seedlings derived from occasional Corinto Nero normal seeds revealed that the few Corinto Nero functional gametes are mostly unreduced. Moreover, three genotypes, including Sangiovese and Corinto Nero, were unexpectedly found to develop fruits without pollen contribution and occasionally showed normal-like seeds. Five missense single nucleotide polymorphisms were identified between Corinto Nero and Sangiovese from transcriptomic data.

**Conclusions:**

Our observations allowed us to attribute a seedlessness type to some variants for which it was not documented in the literature. Interestingly, the *VvAGL11* mutation responsible for Sultanina stenospermocarpy was also discovered in a seedless mutant of Gouais Blanc. We suggest that Corinto Nero parthenocarpy is driven by pollen and/or embryo sac defects, and both events likely arise from meiotic anomalies. The single nucleotide polymorphisms identified between Sangiovese and Corinto Nero are suitable for testing as traceability markers for propagated material and as functional candidates for the seedless phenotype.

**Supplementary Information:**

The online version contains supplementary material available at 10.1186/s12870-021-02865-2.

## Background

Fruit set is defined as the transition of a quiescent ovary to a rapidly growing young fruit [1]. The decision of whether or not to set fruit generally depends on the successful completion of pollination while fruit further growth is determined by fertilization, which initiates seed development [2, 3]. In the absence of pollination and fertilization, the ovary ceases cell division and abscises. Exceptions are parthenocarpic species or varieties within a species, for which the ovary is able to develop in the absence of fertilization, giving a seedless fruit. Parthenocarpy could be attractive to farmers, because it may circumvent the environmental constraints on pollination and fertilization. At the same time, seedless fruits are favourable to both food processing industry and fresh consumption. The wide occurrence of parthenocarpy in fruit crops is likely the effect of a selective pressure for seedlessness during their domestication and breeding [4, 5].

In grapevine, seedlessness is one of the most prized quality traits for table grapes, as demonstrated by the increasing world demand for seedless varieties [6]. Seedlessness might also contribute to a lower cluster density enhancing resilience to pathogen infections [7, 8] and allowing to harmonize ripening periods among berries. In addition, parthenocarpic grapes could ensure a more stable yield over the years, especially in view of climate change [9, 10], when extreme temperatures (heat and cold) and rainy conditions can impair pollen grain and ovule fertility [11, 12]. When related to a limited but still adequate fruit set, the absence of seeds might have favourable effects also on wine quality. A high seedless berry proportion in total berry weight has been found to positively affect wine characteristics (color, taste and aroma) by modifying the berry skin/pulp ratio and avoiding the unpleasant astringency conferred by tannins from immature seeds [13, 14].

Two kinds of seedlessness are reported in grapevine: parthenocarpy and stenospermocarpy [15, 16]. By parthenocarpy, truly seedless berries are produced. In stenospermocarpy, in contrast, ovule fertilization takes place but embryo and/or endosperm abort while the ovule integuments continue to grow to a certain point before stopping. The earlier breakdown occurs, the smaller and more rudimental seed traces are present in the mature berry.

Parthenocarpy is mainly observed in a group of cultivars whose prominent representative is ‘Black Corinth’ or ‘Black Currant’ (alias Korinthiaki). The vast majority of their berries completely lack seeds, are very small and spherical; their use is chiefly to make raisin. Molecular analysis has elucidated that parthenocarpic Corinth type cultivars, including Black Corinth, White Corinth (with a pink variant named Red Corinth), Cape Currant and Corinto Bianco, are not genetically related [17, 18]. In line with this, different reproductive defects have been observed in the above varieties, concerning ovules, embryo sacs and pollen [15, 16, 19–21].

Stenospermocarpy is characteristic of an ancient oriental cultivar known as ‘Kishmish’ (Sultanina or Thompson seedless in the western countries). This variety shares the name Kishmish (or similar) with others often derived from it, and with different genotypes usually of oriental origin [22, 23]. Sultanina has been the major source of seedlessness in table grape breeding programs around the world [17, 24]. Stenospermocarpic berries contain partially developed seeds or seed traces so that are generally considered seedless for commercial purposes; their size, although small, is compatible with requirements for fresh fruit consumption and can be increased by hormone sprays.

The genetic determinism of seedlessness was investigated in both parthenocarpic and stenospermocarpic grapevines.

Parthenocarpy has been recently related with impaired meiosis that terminates in the lack of a mature embryo sac and in pollen sterility in Corinto Bianco, a seedless variant of Pedro Ximenez [21]. At the genomic level, single-nucleotide polymorphisms (SNPs) distinguishing these two lines were identified, among which seven specific to Corinto Bianco were proposed as candidate parthenocarpy-responsible mutations [21]. To our knowledge, no other study has been undertaken to unveil the molecular bases of parthenocarpic phenotype in other cultivars/variants, where independent somatic mutations affecting sexual reproduction are expected.

The genetic architecture of Sultanina stenospermocarpy has been in contrast extensively investigated. In 1996, [25] proposed that three independent recessive genes, which are regulated by a major dominant inhibitor locus named *SDI* (*Seed Development Inhibitor*, according to [26]), control seed development. Different QTL (quantitative trait locus) studies located *SDI* on linkage group (LG) 18, explaining up to 70% of the phenotypic variance in seed content [27–31]. Based on genetic linkage and putative homology, the seed morphogenesis regulator gene *AGAMOUS-LIKE 11* (*VvAGL11*) was proposed as the *SDI* candidate gene [29, 30]. Recent integrative genetics and genomics approaches revealed a missense polymorphism (a SNP at position chr18:26,889,437 resulting in an Arg197Leu substitution) in *VvAGL11* as the functional mutation leading to seed abortion in all Sultanina-related seedless table grape varieties [32]. In the last two decades (since [33] to [34]) , a number of other genes have been proposed to play a role in stenospermocarpic ovule/seed abortion or in normal seed development. Nonetheless, the differential expression detected for these genes in the comparison of seeded and seedless whole fruits might be a consequence (instead of a cause) of the seedless syndrome (with the concurrent lower proportion of seed-related tissues) if these transcripts accumulate specifically in seeds [32]. Additional candidate genes were identified based on the association between structural variations and seedlessness (e.g. [35]).

Despite the efforts made and the positive scientific advances, seedlessness in grapevine remains a phenomenon to be further investigated, especially in respect to new sources of seedlessness. In addition to scientific speculation, such studies could also reveal practical interest in breeding of table grapes as well as of wine grapes.

The present study was undertaken to provide new insights into the regulation of seed and fruit formation in grapevine comparing at phenotypic and molecular levels a set of seedless variants and their seeded counterparts. The mechanisms causing somatic variation in grapevine may include changes in disease (e.g. virus load), epigenetic differences, genetic alterations, or various combinations of these effects [36]. In perennial plant species, where mutants are difficult to generate and to screen, natural somatic variants represent a unique resource to understand the genetic control of target traits, because they may result from the effect of single mutation or epimutation events in a given genetic background [36, 37]. Somatic variants affecting primary berry features like color, seedlessness, or aroma have been identified and exploited throughout the history of viticulture [38]. In the present study, we examined eight pairs of somatic variants with contrasting seed content. All the genotypes investigated are ancient cultivars that are known for having many clonal variants. A high level of somatic variation could have a genetic basis (e.g. a more unstable genetic background) or simply reflect a longer history of cultivation or a larger extension of growth, with consequent remarkable number of vegetative propagation cycles.

Sangiovese has a long-standing documented history, as demonstrated by its first mention in 1590 in Soderini’s treatise “La coltivazione delle viti”. With its 71,558 ha under vines [39], Sangiovese is at present the mostly cultivated grape in Italy and is the basis for the production of renowned wines such as Chianti, Brunello di Montalcino and Vino Nobile di Montepulciano. Sangiovese shows a great phenotypic heterogeneity and is the cultivar with the highest number of registered clones (128) in the Italian National Catalogue of Grape Varieties [40]. The Sangiovese seedless somatic variant evaluated in our study, wrongly named Corinto Nero, was originally identified in the Librandi winery collection in Calabria, South Italy [41]. Other accessions of Corinto Nero with Sangiovese DNA profile were also recovered from Aeolian Islands in Sicily, where in the past they gave the local raisins and are used nowadays in very small proportion in the local wine blend [42]. Further seedless variants of Sangiovese were observed in other Italian regions. The so-called Termarina Nera from Emilia Romagna was included in this study. Other Sangiovese seedless variants are present in Campania (Southern Italy) as Acinella (small berry) (Antonella Monaco, personal communication) and in Piedmont. The appellation “Corinto Nero” refers to a phenotype resembling Korinthiaki (syn. Black Corinth in California/Corinthe Noir in France), the well-known parthenocarpic cultivar from Greece.

Moscato Bianco (syn. Muscat à Petits Grains Blancs) is considered one of the founders of the Muscat family [43]. In the VIVC database [44, 45] it has 327 synonyms. A red parthenocarpic variant of Muscat à Petits Grains Blancs is reported by [17] under the name of “Cape Currant”. The seedless form presented in this study was identified as single sport in a commercial vineyard in Piedmont.

Gouais Blanc (syn. Heunisch Weiss; Liseiret in the present study), the genitor of hundreds traditional grape cultivars, has been cultivated since ancient times in nearly all the temperate European grape growing countries [46] due to its high crop and resistance to cold. Indeed, considerable morphologic variability of Heunisch Weiss clones has been described. Moreover, a stenospermocarpic variant was identified at the JKI Institute for Grapevine Breeding Geilweilerhof, wrongly mentioned by the historic German ampelographers as “Aspirant” [47]. The accession analyzed in the present work corresponds to this variant.

Chasselas, largely grown in central Europe, is an ancient grape cultivar that includes an impressive number of synonyms and sports, with a seedless form named “Chasselas Apyrène” investigated in this study [24].

The stenospermocarpic variety Sultanina is also a very ancient variety subject to somatic variation. Mutants with smaller berries and no abortive seed (“parthenocarpic” Sultanina) [48] or with larger roundish berries and greater seed traces (Sultanina “Gigas”) [49] have been observed. Seeded somatic variants have been also reported as “Thompson seeded” [33] or “Sultanine Monococco” [44, 45]. Dastatchine has been mainly described as a female putative ancestor/offspring of Sultanina [17] but also as an accession of Sultanine Monococco [50]. Here we analyzed as Dastatchine an accession of Sultanine Monococco, i.e. the seeded variant of Sultanina, not its genitor or progeny.

Corinto Bianco was previously reported as a parthenocarpic variant of the ancient seeded cultivar Pedro Ximenez [18], while Termarina proved to be a parthenocarpic variant of the seeded cultivar Sciaccarello (syn. Termarone) [51]. We also included in our analysis Corinthe Noir (syn. Korinthiaki/Black Corinth) as a reference for parthenocarpy.

With the exception of Corinto Bianco and Sultanina, the basis of the variation in seed and fruit formation in the other variants has not been investigated so far. Sangiovese and its seedless variant called Corinto Nero were studied here in more detail.

## Results

### Genotyping variant pairs

In order to assess the possibility of distinguishing somatic variants from the same cultivar, each pair of clones (Table 1) was genotyped with SSRs (Simple Sequence Repeats) and SNPs.

**Table 1:** List of accessions analyzed in the present work

Variety^**a**^	Seeded variant accession name	Seedless variant accession name	Germplasm collection^**b**^	Reference for previous identification or characterization	Ascribed seedlessness type
Sangiovese	Sangiovese (clone R24)	Corinto Nero	Grinzane Cavour Grugliasco San Michele a/A	[41, 52]	Parthenocarpy
		Termarina Nera	Grinzane Cavour	-	-
Gouais Blanc (Heunisch Weiss)	Liseiret	Aspirant-false	Grinzane Cavour	[47]	Stenospermocarpy
Moscato Bianco (Muscat à Petits Grains Blancs)	Moscato Bianco	Moscato Bianco seedless (mutant)	Grinzane Cavour	[17]	Parthenocarpy
Sciaccarello	Termarone	Termarina Rosa	Grinzane Cavour	[51]	Parthenocarpy
Chasselas	Chasselas^c^	Chasselas apyrène	San Michele a/A	[24]	Stenospermocarpy
Sultanina	Dastatchine-false (Sultanine Monococco)	Sultanina (Bianca)	San Michele a/A	[17, 32]	Stenospermocarpy
Pedro Ximenez	Pedro Ximenez	Corinto Bianco	San Michele a/A	[18, 21]	Parthenocarpy
Korinthiaki	-	Corinthe Noir	San Michele a/A	[20, 53]	Parthenocarpy

^a^The variety name was given to each accession according to the true-to-type SSR profile match found in the *Vitis* International Variety Catalogue [44, 45] and/or the European Vitis Database [50]

^b^Plants in Grinzane Cavour and Grugliasco are managed by CNR-IPSP, plants in San Michele a/A by FEM

^c^Blanc for genotyping and for emasculation trials, Rose for phenotyping (there were no available Chasselas Blanc plants in the FEM germplasm collection in 2018)

The accessions belonging to each pair shared the same microsatellite profile (Additional file 1: Table S1). Liseiret/Aspirant proved to be identical to Heunisch Weiss, synonym Gouais Blanc [47], Corinthe Noir to Korinthiaki, synonym Black Corinth [18] and Termarone/Termarina Rosa to Sciaccarello, synonyms Mammolo/Verano/Termarina [51].

After SNP dataset filtering, pairwise analysis revealed identical SNP profile at all the passed loci of the GrapeReSeq_Illumina_20K_SNP_chip for Sangiovese/Corinto Nero, Termarone/Termarina Rosa, Chasselas Blanc/Chasselas apyrène and Pedro Ximenez/Corinto Bianco. Potentially different SNPs between the other somatic variants were not confirmed by Sanger sequencing of PCR products (data not shown).

In conclusion, the clones analyzed here could not be differentiated using microsatellites and SNPs from the GrapeReSeq_Illumina_20K_SNP_chip.

### Phenotyping variant pairs upon open-pollination

#### Seed formation, fruit set and berry/bunch features

Given that the eight somatic variants under study were identified based on their impaired seed formation, they were first compared to their original varieties in terms of seed content. Additional traits were evaluated as being potentially affected by seed formation. In particular, the level of seed formation and development can impact on fruit set and berry size; those in turn affect bunch development, weight, size and density.

The differential behavior of seedless and seeded variants was stable across seasons/locations for most traits (Additional file 2: Fig. S1).

**Fig. 1:**
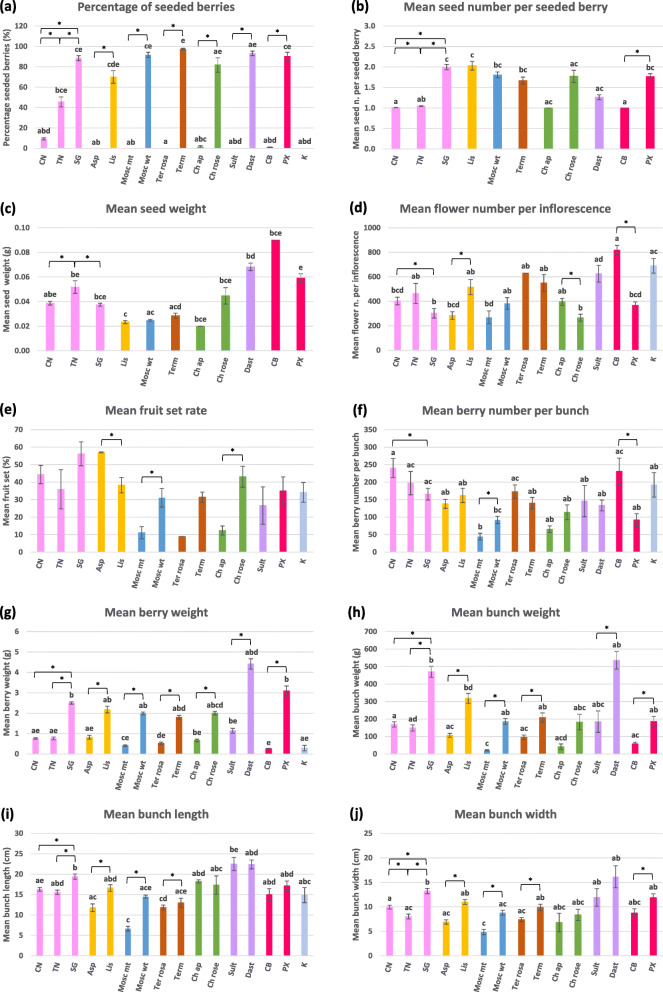
Phenotyping of variant pairs upon open-pollination. Members of the same pair (or triplet) are depicted with the same color. For each accession, a mean value was calculated from different bunches, seasons and locations. Bars correspond to standard errors. Asterisks indicate significant (*P* < 0.05) differences between seeded and seedless variant pairs, as established by one or more test(s) among T-Student test (or Welch test in the case of unequal variances), Mann-Whitney test, and Kolmogorov-Smirnov test. Different letters indicate significant differences in the whole set of accessions (Kruskal-Wallis test followed by Dunn’s post hoc test with Bonferroni correction for multiple tests, *P* < 0.05). In (**a**) berries with apparently normal seeds were considered as seeded, whereas berries containing only rudimental seeds, seed traces or unfertilized ovules were classified as seedless. In (**e**) Corinthe Noir fruit set rate measured at harvest (34%) was lower than that evaluated at fruit set stage (68%), since most berries were dried and part of them had already fallen. Abbreviations: CN = Corinto Nero, TN = Termarina Nera, SG = Sangiovese, Asp = Aspirant-false, Lis = Liseiret, Mosc mt = Moscato Bianco mutant, Mosc wt = Moscato Bianco, Ter rosa = Termarina Rosa, Term = Termarone, Ch ap = Chasselas apyrène, Ch rose = Chasselas Rose, Sult = Sultanina, Dast = Dastatchine-false, CB = Corinto Bianco, PX = Pedro Ximenez, K = Corinthe Noir (reference for parthenocarpy)

**Fig. 2:**
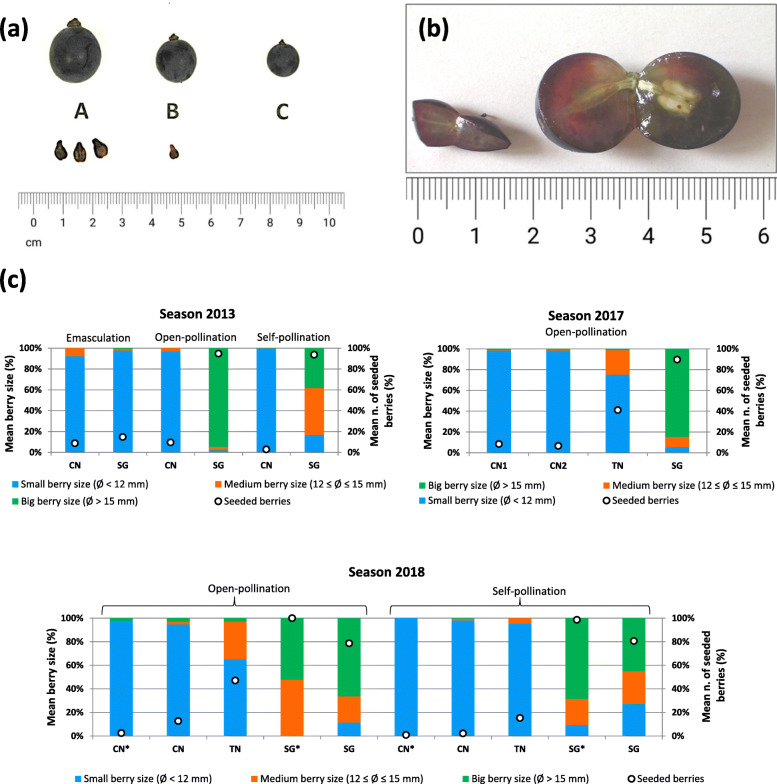
Relationship between berry size and presence of normal seeds in Sangiovese and its seedless variants. (**a**) Classification of berries according to size; the prevalent type of seeds or seed traces is shown at the bottom. (**b**) Representative berries from Corinto Nero (on the left) and Sangiovese (on the right). (**c**) Percentage distribution of berries according to size and seed content. The percentage of small, medium and large berries was calculated from the total number of berries per bunch, while the percentage of seeded berries was established on the total number of berries opened for seed examination (it was a representative portion of the total number of berries when this number was too big). Berries with apparently normal seeds were considered as seeded, whereas berries containing only rudimental seeds, seed traces or unfertilized ovules were classified as seedless. For each combination of accession, season and pollination treatment, from one to nine clusters were analyzed and an average value was calculated. Abbreviations: CN = Corinto Nero, TN = Termarina Nera, SG = Sangiovese in the Grinzane Cavour collection (Corinto Nero plants from two distinct parcels were analyzed in 2017); CN*, SG* = Corinto Nero, Sangiovese in the FEM collection, respectively

As expected, all the seedless clones had a significantly lower percentage of seeded berries compared to their wild-type counterparts, according to 2 years of analysis (2017 and 2018) for most pairs (Fig. 1a and Additional file 1: Table S2). In particular, Aspirant, Moscato Bianco mutant, Termarina Rosa, Sultanina and Corinthe Noir proved to be absolutely devoid of normal seeds (however, a few seeded berries, which were also bigger than normal, were noticed in Moscato Bianco mutant and Corinthe Noir in 2019, as described in the section “Inspection of seeds and traces of reproductive structures at veraison”). For the other seedless lines, the proportion of seeded berries ranged from 1% (in Corinto Bianco) to 45.6% (in Termarina Nera) (Additional file 1: Table S2). For Corinto Nero the average percentage of seeded berries was 9.3%, which is consistent with the values previously calculated from a greater number of berries (5.0, 3.1 and 4.3% of seeded berries out of 2133, 1539, 1456 total berries collected in 2008, 2009 and 2010 respectively). It can be easily noticed that the two seedless variants of Sangiovese, Corinto Nero and Termarina Nera, show a rather different phenotype with a higher percentage of medium-sized berries and seeded berries in the last one especially when subjected to open-pollination (Fig. 2c). The seeded berries present in the seedless accessions displayed a comparable size to that of berries from their seeded counterparts (data not shown).

Seeded berries from seedless accessions contained one apparently normal seed on average (Fig. 1b and Additional file 1: Table S2). In particular, all seeded berries from Chasselas apyrène and Corinto Bianco showed one seed, while a few seeded berries from Corinto Nero and Termarina Nera had a second seed. However, the majority of these seeds are not expected to be viable, as suggested by empty seed rate (data not shown). For example, this rate (as estimated by floatability) proved to be more than 11-fold higher in seeded berries from Corinto Nero (72.3%) compared to Sangiovese (6.3%) (Table 2). Seeded berries from seeded accessions accommodated from one to two normal seeds on average. Among seeded lines, the minimum and the maximum number of seeds were observed in Dastatchine and in Liseiret/Sangiovese, respectively (Fig. 1b and Additional file 1: Table S2). The majority of fully developed seeds were found in large berries (class A), as shown in Fig. 2 and in Additional file 3: Fig. S2. The mean seed fresh weight was not significantly different between Corinto Nero and Sangiovese seeded berries, while Termarina Nera seeded berries contained significantly heavier seeds. Among the seeded varieties, Liseiret and Moscato Bianco showed the lightest seeds, whereas Dastatchine had the heaviest ones, which suggests a negative relationship between seed number and weight in these genotypes (Fig. 1c and Additional file 1: Table S2).

**Table 2:** Number of seeds that were recovered from open-pollinated Corinto Nero berries in 2016 and *in vitro* germinated to generate plantlets for ploidy level analysis and genotyping. Seedlings were obtained from 50 non-floating Sangiovese seeds with the only objective to calculate their germination rate

	Total seeds	Normal seeds (non-floating in water)	Potentially viable seeds (%)	Seeds cultivated *in vitro*	Germinated seeds	Germination rate (%)
Sangiovese (clone R24)	429	402	93.7	50	27	54.0
Corinto Nero	629	174	27.7	165	85	51.5

**Fig. 3:**
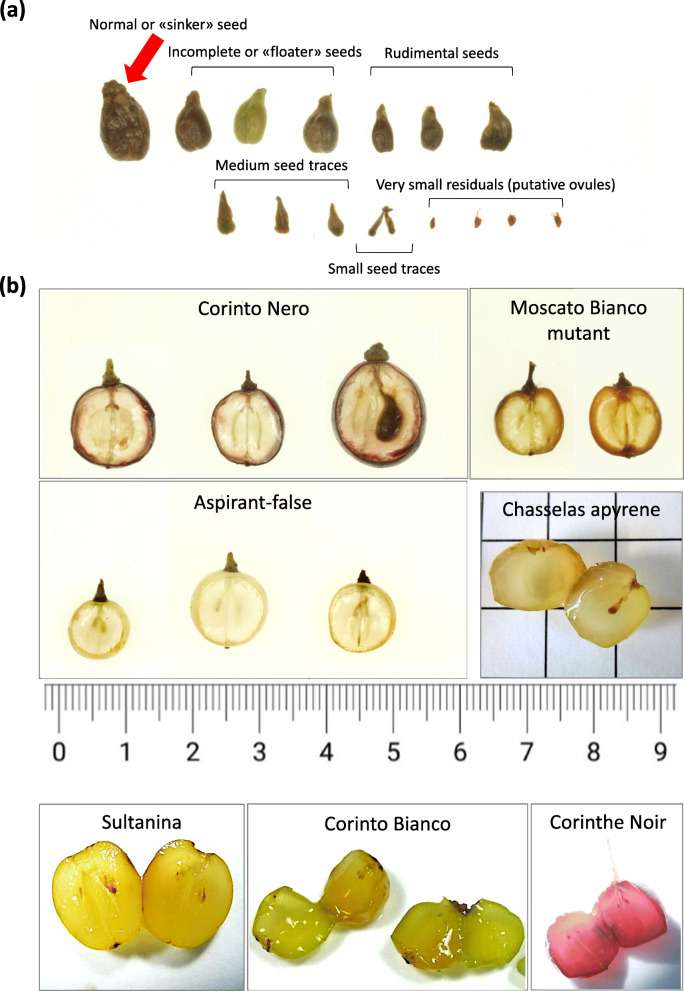
Seed evaluation. (**a**) Gradient of seed development observed in the accessions under study. Only normally developed seeds (as indicated by the arrow) were considered to estimate the percentage of seeded berries. They possess a normal testa (consisting of outer and inner integument), endosperm and embryo. The remaining structures are supposed to correspond to incomplete (“floater”) or rudimental seeds, seed traces and ovules. (**b**) Sections of berries from the seedless lines considered in this work. The rightmost Corinto Nero berry contains a normal seed

In addition or as an alternative to normally developed seeds, various rudimental seeds, seed traces and tiny residuals (likely ovule traces) were found in most cases (Fig. 3). For the Sangiovese/Corinto Nero case study, in 2018 we quantified the proportion of seeded berries, berries with only traces (attributed to ovules later in the text) and berries devoid of any rudiment, as shown in Additional file 4: Fig. S3. Upon open-pollination, all Sangiovese berries contained at least one apparently normal seed, while the majority of Corinto Nero berries were devoid of any rudiment, a smaller percentage contained traces and only 2.5% accommodated a seed.

In order to assess the possible impact of seed development on fruit development, each seedless variant was compared to its original variety in terms of fruit set rate. This parameter was only evaluated in 2018, for which reason the data reported below cannot be considered as conclusive. As a preliminary result, we verified that there were no significant differences between fruit set rate measured at fruit set stage and at harvest (data not shown). Except for Aspirant, all seedless variants showed lower fruit set rates (as estimated at harvest) than their seeded counterparts, with statistically significant differences observed for all the analyzed pairs but Corinto Nero/Sangiovese and Termarina Nera/Sangiovese (Fig. 1e and Additional file 1: Table S2). Nonetheless, differences in fruit set rate between Corinto Nero and Sangiovese were significant in self-pollination conditions at IPSP (data not shown). Fruit set could not be figured for Dastatchine due to the low number of available inflorescences and for Corinto Bianco because all inflorescences dried after flowering.

The two factors determining fruit set rate, which are the number of flowers per inflorescence and the number of berries per bunch, were further investigated. Significant (*P* < 0.05) differences in the average flower number per inflorescence were found in several seedless/seeded pairs (Fig. 1d and Additional file 1: Table S2). In particular, the seedless variants Corinto Nero, Chasselas apyrène and Corinto Bianco showed a significantly greater number of flowers per inflorescence with respect to their seeded counterparts. Although we could not perform any statistical comparison due to the missing seeded reference (Dastatchine did not produce enough inflorescences), it was evident that additional seedless accessions (Sultanina and Corinthe Noir) exhibited a high flower number. This general behavior was inverted for Aspirant that had a significantly lower number of flowers than Liseiret. Similarly, berry number per bunch did not show a clear pattern related to seed content and it appeared instead to be genotype-dependent. In particular, the seedless variants Corinto Nero and Corinto Bianco exhibited a significantly greater number of berries per cluster with respect to their seeded counterparts. This trend was inverted for the seedless Moscato Bianco that had a significantly lower number of berries than its wild-type (Fig. 1f and Additional file 1: Table S2).

More evident was the impact of seed formation on fruit size and bunch features, as revealed by 2 years of analysis (2017 and 2018) for most pairs and up to four in the case of Corinto Nero/Sangiovese.

**Fig. 4:**
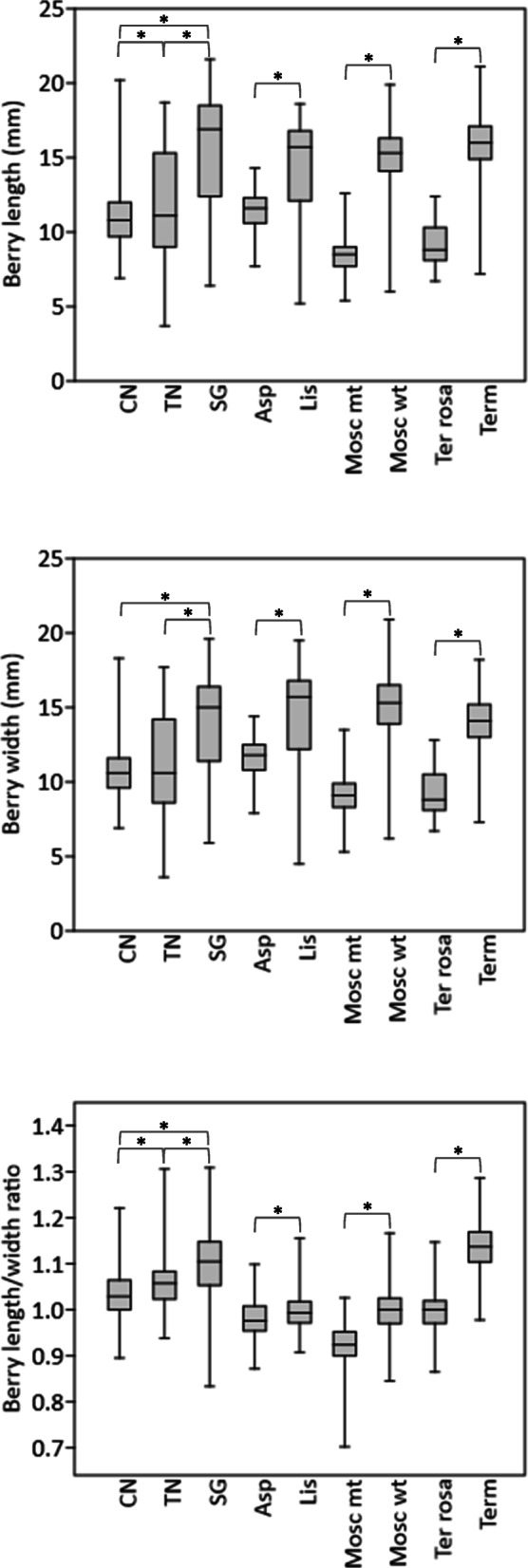
Berry evaluation. Berry size and shape were evaluated with a digital caliper in 2017 and 2018 (for the pair Aspirant/Liseiret data were registered only in 2017). When more than 50 berries per bunch were available from one berry size category, pictures were taken from 50 berries; when there were less than 50 berries per bunch belonging to a size category, pictures were taken from all berries. The number of analyzed berries ranged from a minimum of 280 (Moscato Bianco mutant) to a maximum of 1137 (Corinto Nero). The 25–75% quartiles are shown with a box, the median with a horizontal line inside the box, the minimal and maximal values with short horizontal lines (“whiskers”). Asterisks indicate significant (*P* < 0.05) differences between seedless and seeded variant pairs, as established by Mann-Whitney test. Abbreviations: CN = Corinto Nero, TN = Termarina Nera, SG = Sangiovese, Asp = Aspirant-false, Lis = Liseiret, Mosc mt = Moscato Bianco mutant, Mosc wt = Moscato Bianco, Ter rosa = Termarina Rosa, Term = Termarone

Berries from all seedless accessions proved to be significantly lighter compared to berries from the corresponding seeded clones (Fig. 1g and Additional file 1: Table S2). In the set of IPSP accessions (where both berry length and width were measured), berries from seedless lines were shorter and narrower than berries from seeded lines and, as a general trend, they had a more rounded shape (Fig. 4). These data confirm the existence of a significant correlation (R = 0.79) between mean berry weight and mean seed number per berry. Mean berry weight proved to be significantly correlated (R = 0.67) also with mean seed weight only in the pool of seeded accessions. For example, Dastatchine (and Pedro Ximenez to a lesser extent) had both the heaviest seeds and the heaviest berries.

**Fig. 5:**
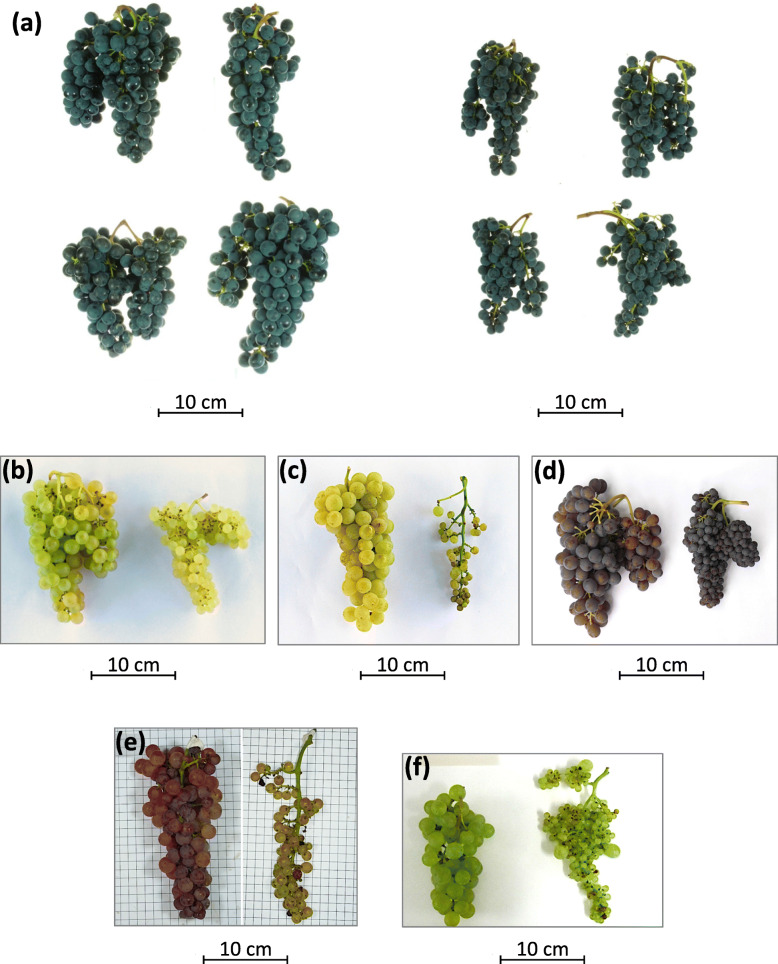
Bunch evaluation. Comparison between (**a**) Sangiovese and Corinto Nero, (**b**) Liseiret and Aspirant, (**c**) Moscato Bianco wild-type and mutant, (**d**) Termarone and Termarina Rosa, (**e**) Chasselas Rose and Chasselas apyrène, (**f**) Pedro Ximenez and Corinto Bianco clusters deriving from open-pollination. In each picture, the seeded cultivar is shown on the left, the seedless variant on the right

As a rule, clusters from seedless variants were significantly lighter than clusters from the corresponding seeded lines (Fig. 1h and Additional file 1: Table S2). In most cases, they were also shorter and narrower, with a greater length/width ratio (Fig. 1i-j, Fig. 5 and Additional file 1: Table S2). The majority of seedless variants had looser bunches compared to their seeded counterparts (according to the OIV 204 descriptor and to one or, more often, 2 years of evaluation). The most evident exception was Termarina Rosa (Additional file 1: Table S3). In line with this, when considering all the accessions in the same analysis, bunch compactness proved to be positively correlated with seed content (percentage of seeded berries and number of seeds per berry), fruit set rate, berry size (weight) and bunch size (weight, length and width, as well as the ratio between weight and size). Most of the genotypes had a similar relationship between bunch compactness and the above traits, with the only exception of Termarone (alias Sciaccarello), Termarina Rosa wild-type. When performing a separate analysis for each genotype, an additional positive correlation was found between bunch compactness and berry number (as well as the ratio between berry number and bunch length), which can justify the use of berry number as an indicator of bunch compactness (Additional file 1: Table S4).

In conclusion, our collective data indicate that the level of seed development has a potential impact on fruit set rate and a significant effect on berry/bunch features.

#### Inspection of seeds and traces of reproductive structures at veraison

In order to establish the type of seedlessness occurring in each seedless variant, it is essential knowing the nature of the traces found in seedless berries: unfertilized ovules (parthenocarpy) or seeds aborted at some point after fertilization (stenospermocarpy). To this purpose, in 2019 we inspected in all genotypes (but three) seeds and traces at veraison. Indeed, at this phenological stage grapevine seeds have already reached their full pear shape and size (from a structural point of view, they are completely developed) and traces can be more easily extracted and analyzed than at maturity.

Two different berry size categories (small and large) were observed for all the seedless accessions but Sultanina, and only in Liseiret among the seeded ones (Table 3).

**Table 3:** Quantitative and qualitative description of the traces (of ovules or seeds) and the seeds extracted from the berries inspected at veraison for the investigated genotypes

Accession	Berry size	N of berries	Berry diameter range (mm)	N of traces	N of seeds	N of potentially vital seeds^**a**^	Traces per berry	Seeds per berry	Observations
Corinto Nero	Small	10	8.2 - 10.2	16	0	-	1.6	0	Very small traces. They were very soft, impossible to dissect because they fell apart. Traces completely absent in two berries out of ten
Corinto Nero	Large	10	13.0 - 16.3	13	10	10	1.3	1.0	Despite all seeds were potentially vital, when dissected, some of them presented a degenerated endosperm. An embryo was observed in some seeds with a well-developed endosperm
Sangiovese	Unique	10	12.2 - 14.2	0	24	24	0	2.4	
Aspirant	Small	10	NA	23	0	-	2.3	0	Some traces were completely soft, while others showed a partially developed sclerenchyma
Aspirant	Large	10	NA	29	0	-	2.9	0	In general, traces extracted from the biggest berries were more developed than those from smaller berries
Liseiret	Small	10	NA	14	18	12	1.4	1.8	
Liseiret	Large	10	NA	10	33	22	1.0	3.3	
Moscato Bianco mutant	Small	10	7.0 - 8.4	0	0	-	0	0	Berries with no traces at all
Moscato Bianco mutant	Large	2	13.8 - 17.0	0	4	4	0	2.0	Seeds presented a well-developed endosperm
Moscato Bianco	Unique	10	13.0 - 16.6	0	19	19	0	1.9	
Termarina Rosa	Small	10	7.0 - 8.2	32	0	-	3.2	0	Very small and soft traces, impossible to dissect because they were destroyed due to the reduced size and fragility
Termarina Rosa	Large	10	8.2 - 9.9	24	0	-	2.4	0	Very small and soft traces, impossible to dissect because they were destroyed due to the reduced size and fragility
Termarone	Unique	10	14.0 - 16.2	0	29	28	0	2.9	
Chasselas apyrène	Small	10	11.2 - 14.1	27	0	-	2.7	0	Soft traces, heterogeneous in size. Some of them, despite being soft and with no well distinguishable structure inside, had the appearance of a seed on a reduced scale
Chasselas apyrène	Large	10	14.5 - 16.8	8	15	4	0.8	1.5	The potentially vital seeds contained a well-developed endosperm. The seeds that floated where hollow inside, when dissected only seed coat and sclerenchyma structures were observed, while endosperm was usually not present (nor embryo) or was partly degenerated
Sultanina	Unique	10	11.2 - 14.2	29	0	-	2.9	0	Soft traces, heterogeneous in size. A seed shape (pear shape) was observed for the biggest ones, which could also be longitudinally or transversally dissected. No sclerenchyma was formed and inside a very thin white tissue with an aqueous or gel-like consistency could be distinguished
Corinthe Noir	Small	11	7.5 - 9.9	19	0	-	1.7	0	Traces were tiny and soft, it was impossible to dissect them because they were destroyed due to their reduced size and fragility
Corinthe Noir	Large	3	12.5 - 17.3	5	4	1	1.7	1.3	Traces were similar to those of the smaller berries. Almost all inspected seeds were empty. One out of the four seeds presented an endosperm, but no embryo was observed

*Abbreviations*: *N* number, *NA* not analyzed

^a^Based on the floatation test: seeds that sank into water were considered potentially vital

First, the biggest berries of the seedless genotypes and all the berries of the seeded genotypes were inspected for the presence of seeds. In all the dual-sized seedless variants but Aspirant and Termarina Rosa, the few recovered large berries contained seeds (Table 3 and Additional file 5: Figures S4-S8). The floatation test suggested that the seeds of Corinto Nero and Moscato Bianco mutant were vital, whereas the majority of those of Chasselas apyrène and Corinthe Noir were not. When potentially viable seeds were dissected, a well-developed endosperm was usually observed, while the embryo was not. This is probably due to the type of section performed, thus the presence of an embryo cannot be excluded. Aspirant biggest berries accommodated only traces of reproductive structures, but initiation of seed components could be generally observed in a more advanced stage of development than in smaller berries (Additional file 5: Figure S4). In the case of Termarina Rosa, large berries showed instead traces similar to those contained in small berries (Additional file 5: Figure S7a-c). Unlike the other seedless variants, berry size differences in Aspirant and Termarina Rosa are probably due to a phenological lag between berries sampled from different parts of the bunch or from different bunches. By the time of harvest, all the berries would have likely reached a homogenous size. In fact, this was also observed for Aspirant seeded counterpart (Liseiret), whose small and large mature berries presented well-developed seeds. Detailed description of the seeds extracted from each seeded genotype is shown in Additional file 5: Figure S9. Significant differences were found in seed length and width in the seedless/seeded pairs analyzed, that are Corinto Nero/Sangiovese and Moscato Bianco mutant/Moscato Bianco (Additional file 1: Table S5). It is noteworthy that Corinto Nero seeds were on average larger and wider than those of all the other accessions.

**Fig. 6:**
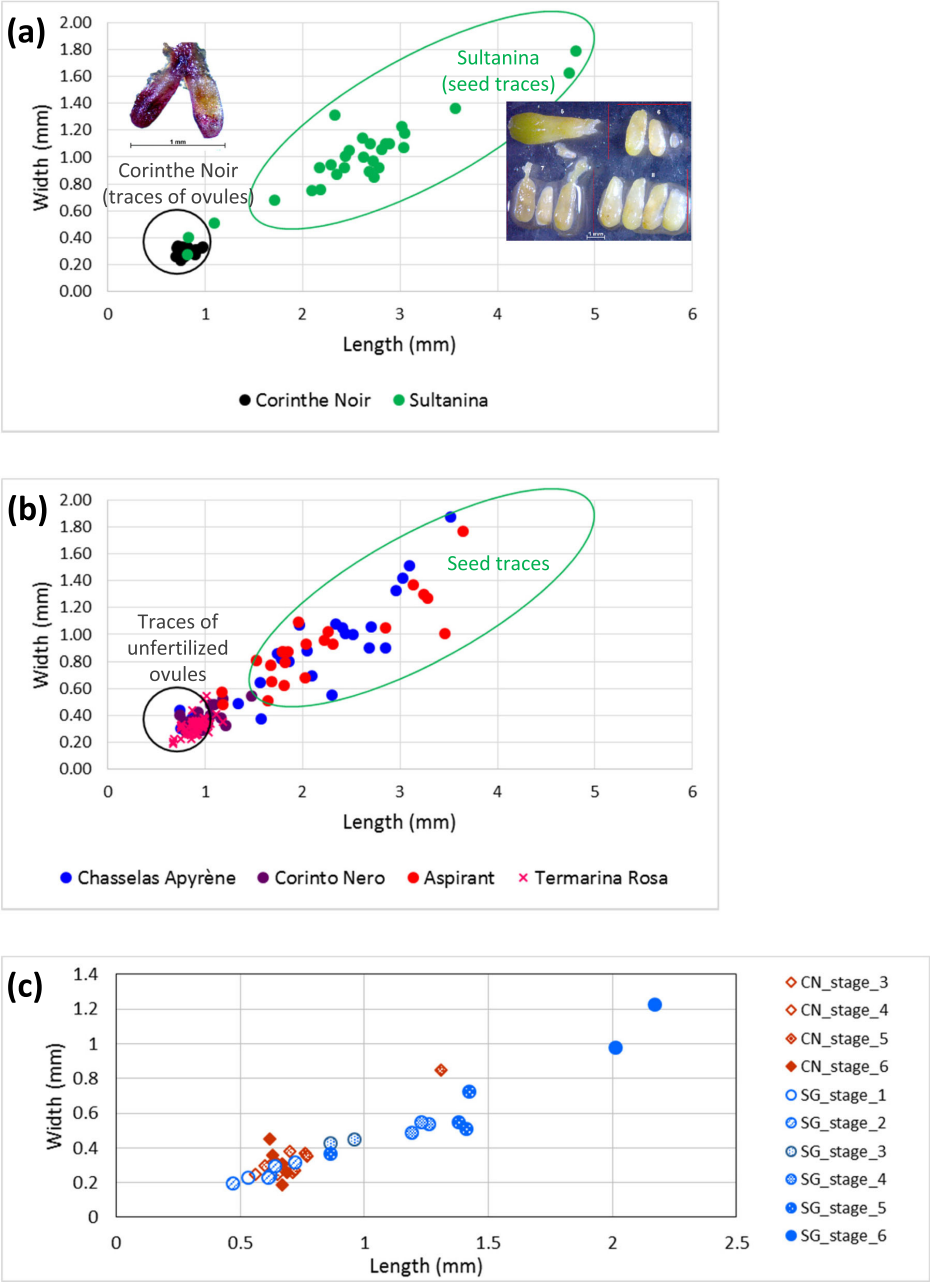
Scatter plots of traces’ length against traces’ width for the reference cultivars for parthenocarpy and stenospermocarpy, Corinthe Noir and Sultanina, respectively (**a**), and for the other seedless accessions under investigation (**b**). Reported measures refer to traces extracted only from the smaller berries (with the exception of Sultanina having berries of a unique size). In (**c**) scatter plot of the length against the width of the ovules/seed traces of Corinto Nero (CN) and Sangiovese (SG) measured at six stages from flowering (stage 1) to pepper-corn size (stage 6), as detailed in Additional file 6: Figure S10. The intensity in the color filling the diamonds/dots increases with the stages. Ovules from stages 1 and 2 of Corinto Nero could not be measured because they were destroyed during extraction from the ovary due to their reduced size and fragility

Then, traces of reproductive structures were inspected in seedless berries of seedless accessions. We assumed that, in case traces were observed in seedless berries of the reference cultivars for parthenocarpy (Corinthe Noir) and stenospermocarpy (Sultanina), they are likely remnants of unfertilized ovules and seed traces, respectively. Soft traces were found in the analyzed berries of these two genotypes (Additional file 5: Figure S8). However, significant differences were detected in their length and width (Additional file 1: Table S6). In particular, traces of Corinthe Noir proved to be much smaller compared to the great majority of traces of Sultanina (Fig. 6a). As regards the other seedless variants that were analyzed, berries of Moscato Bianco mutant contained no traces at all, Corinto Nero and Termarina Rosa traces clustered together with Corinthe Noir ones, whereas Chasselas apyrène and Aspirant traces mainly laid within the size range of Sultanina (Fig. 6b). In fact, significant differences both in trace length and width were found between accessions grouped in the Corinthe Noir cluster (Corinthe Noir, Corinto Nero and Termarina Rosa) and those of the Sultanina’s size range (Sultanina, Aspirant and Chasselas apyrène), but not between accessions within each group (Additional file 1: Table S6). Based on these results, we hypothesize that most of Corinto Nero and Termarina Rosa traces are likely unfertilized ovules, while those found in the seedless berries of Aspirant and Chasselas apyrène are probably remnants of seeds aborted in earlier or later stages of development. Pieces of evidence that fertilization occurs in Aspirant and Chasselas apyrène were also the presence of structures such as sclerenchyma and/or endosperm, a big degenerated nucellus, and a clearly defined pear shape of seed traces extracted from their seedless berries (Additional file 5: Figures S4 and S5). Conversely, none of these structures or characteristic seed shape could be seen in the examined traces from seedless berries of Corinto Nero and Termarina Rosa (Additional file 5: Figures S6 and S7a-c). When analyzed at six stages from flowering to pepper-corn sized berries, the ovules of the Sangiovese seedless variant essentially remained within the same range of length and width, which further confirms the above hypothesis that they are unfertilized ovules. Oppositely, the ovules of Sangiovese wild-type increased in size with the progress of the phenological stages, that is to say, they are likely fertilized ovules evolving to become a seed (Fig. 6c and Additional file 6: Figure S10).

Our collective observations indicate the occurrence of parthenocarpy in Corinto Nero, Moscato Bianco mutant and Termarina Rosa, of stenospermocarpy in Aspirant and Chasselas apyrène.

### Understanding the basis of the variation in seed development

In order to figure out the basis of the variation affecting seed formation in the accessions under study (with special emphasis on Corinto Nero), we investigated one possible driving factor that is gamete functionality.

#### Evaluation of male gamete (pollen) functionality

##### Pollen viability and germination

**Fig. 7:**
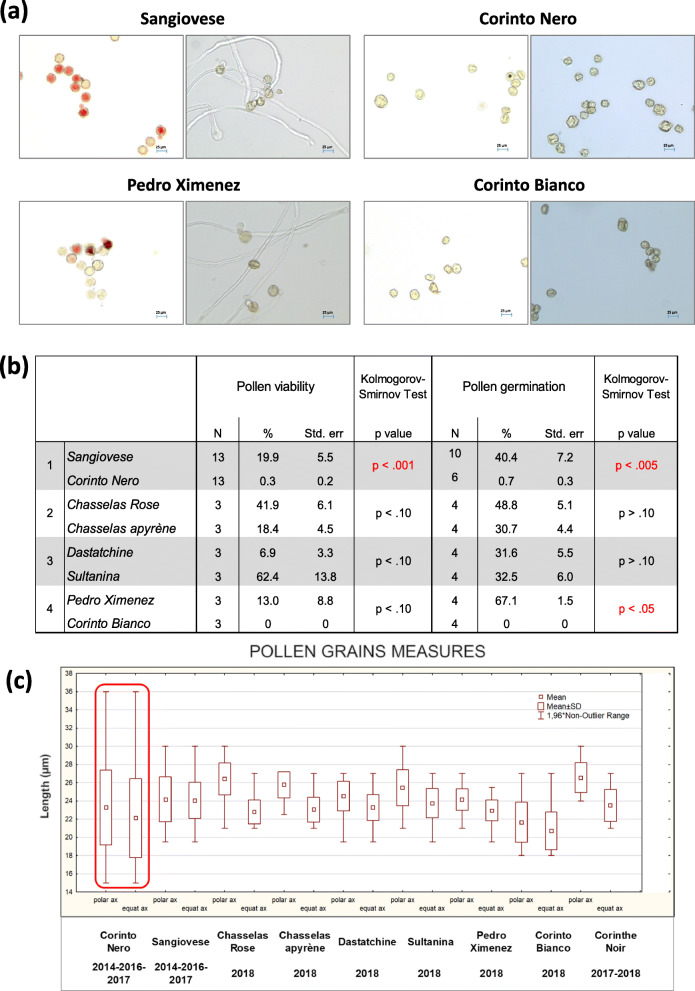
Evaluation of pollen functionality and morphology. (**a**) Pictures of some Sangiovese, Corinto Nero, Pedro Ximenez and Corinto Bianco pollen grains subjected to the viability (on the left) and germination (on the right) in vitro tests, as observed at the microscope (200X). (**b**) Mean values (± standard error) of pollen viability and germination percentage per accession; N is the number of replicates. The total number of observed pollen grains per accession ranged from a minimum of 1040 to a maximum of 4528, in relation to the available inflorescences. To detect differences between each seeded variety and its seedless variant, the non-parametric Kolmogorov-Smirnov test was performed. (**c**) Box plots representing the polar and equatorial axis lengths measured on fifty randomly selected pollen grains for each genotype in each season. Abbreviations: ax = axis, SD = standard deviation, Std. err = standard error

The in vitro viability and germination of Corinto Nero pollen grains proved to be null or close to zero in three seasons (2014, 2016 and 2017). Conversely, Sangiovese pollen viability and germination rates were on average 20 and 40%, respectively. The behavior of Corinto Nero pollen closely resembles that of Corinto Bianco, for which we observed no viability and germination ability in 1 year (2018), while the pollen grains of its seeded counterpart (Pedro Ximenez) showed high germinability instead. Oppositely, both Chasselas apyrène and Sultanina had functional pollen in the same year (Fig. 7a-b). High viability and germination were registered also for Corinthe Noir pollen in two seasons, 2017 and 2018 (with average values of 79 and 44%, data not shown).

##### Pollination treatments

Self- and cross-pollination experiments were carried out to test the in vivo performance of pollen from the seedless variants.

A) Self- vs open-pollination: Pollen source proved to have a different impact on seed formation in Sangiovese and its seedless variants. In particular, while Sangiovese open- and self-pollinated bunches exhibited the same percentage of seeded berries, the fraction of Corinto Nero berries with normal seeds was significantly lower after self-pollination, according to the trials done in 2013 and 2018. Similar results are expected for Termarina Nera, but they are not supported by any statical test since a single self-pollinated bunch was evaluated in 2018. Conversely, out of the analyzed seedless variants that set some seeds in 2018, Chasselas apyrène was not influenced in the percentage of seeded berries by the pollination treatment (Additional file 1: Table S7).

Similarly to what found for seed set, fruit set rate (as estimated in 2018) proved to be significantly reduced in self-pollination conditions for Corinto Nero located at IPSP. Conversely, it did not vary between pollination treatments for the other seedless accessions for which a statistical comparison was feasible (Chasselas apyrène, Sultanina and Corinthe Noir). The same results were obtained for the mean number of berries per bunch, as evaluated in 2013 and/or 2018 (Additional file 1: Table S7).

B) Pollination of Nebbiolo/Trebbiano Toscano with Corinto Nero pollen: Berry set was poor when Nebbiolo was cross-pollinated with Corinto Nero pollen compared with fruit set rate of the self-pollinated inflorescences of Nebbiolo. Similar results were obtained when Trebbiano Toscano was cross-pollinated with Corinto Nero pollen. Almost all seeds obtained from Nebbiolo and Trebbiano Toscano cross-pollination with Corinto Nero pollen did not germinate (Table 4).

**Table 4:** Cross-pollination of Trebbiano Toscano and Nebbiolo with Corinto Nero pollen. Self-pollination was used as a control in the trial involving Nebbiolo

Variety	Year	Self-pollination		Cross-pollination with Corinto Nero pollen		
		Inflorescences that set fruit	Berries and seeds	Inflorescences that set fruit	Berries	Seeds
Trebbiano Toscano	2012	Not performed		3/24	Several berries were observed at fruit set stage. However, at the end of the ripening season only a few berries were still present	Twenty-nine normal seeds were recovered, but none was able to germinate
	2013	Not performed		2/9	The two clusters had small berries	No seed
Nebbiolo	2013	4/4	The ripe clusters had 225 berries on average. Around 74% of the berries contained normal seeds (1.7 seeds per berry, 2.3 seeds per seeded berry on average)	1/10	The ripe cluster had 29 small berries	All the berries contained seeds (1.8 seeds per berry on average). Forty-nine seeds were recovered, but only two germinated
	2014	4/5	The clusters had several berries	0/3		

C) Emasculation of some pairs and additional varieties: This experiment was originally done to evaluate the parthenocarpic potential of Corinto Nero, given that this accession was found to set fruit in self-pollination conditions in spite of having non-functional pollen, and was then extended to other accessions. While the emasculated and covered inflorescences from most of the treated genotypes dried, Sangiovese, Corinto Nero and Gamay proved to set fruit after anther (and, when tested, also stigma) removal. This ability was confirmed in different seasons and locations but Sangiovese lost its ability to set fruit when emasculation/destigmation was performed at the earliest stage (E-L 15), whereas Gamay was apparently not influenced (Table 5). In 2019, the fruit set rate calculated for Sangiovese and Corinto Nero after emasculation was 42 and 21%, respectively (compared to 66 and 50% upon open-pollination) (data not shown).

**Table 5:** Outcome of emasculation trials. Numerator and denominator indicate bunches that set fruit after treatment and covering and starting inflorescences, respectively. Occasional normal seeds from the bunches in boldface were put to germinate and the seedlings were genotyped with microsatellite markers

**Accession**	**Collection**	**Year, stage and pollination treatment**												
		**2012**		**2013**					**2014**					
		Stage II		Stage II					Stage II					
		SP	EMS+ST	SP	EMS+ST		EMS-ST		SP		EMS+OP		EMS+ST^a^	EMS-ST
Sangiovese R24	IPSP, FEM	4/4	4/4	4/4	6/7		1/1		5/5				5/5	1/1
Corinto Nero	IPSP	4/4	5/5	4/4	3/4				5/5		1/1		4/5	1/1
Chasselas Blanc	FEM	2/2	0/4	2/2	0/4									
Chasselas apyrène	FEM	2/2	0/4	2/2	0/4									
Pedro Ximenez	FEM	2/2	0/4	2/2	0/4									
Corinto Bianco	FEM	1/1	0/2											
Nebbiolo	IPSP			4/4	**1/4**				4/5				0/5	
Trebbiano Toscano	FEM		0/1		0/1									
Gamay	IPSP			5/5	**4/5**				4/5				6/6	
Gouais (syn. Liseiret)	FEM				0/4									
**Accession**	**Collection**	**Year, stage and pollination treatment**												
		**2015**					**2016**						**2019**	
		Stage I		Stage II			Stage I			Stage II			Stage II	
		SP	EMS+ST	SP	EMS+ST	EMS-ST	SP	EMS+ST	EMS-ST	SP	EMS+ST	EMS-ST	OP	EMS+ST
Sangiovese R24	IPSP, FEM	**3/3**	0/2	4/4	**3/4**	1/3	1/1	0/5	0/5	**1/1**	**4/5**	2/5	5/5	3/5
Corinto Nero	FEM												5/5	3/7
Nebbiolo	FEM						1/1	0/2	0/2	1/1	0/2	0/2		
Gamay	IPSP, FEM	**3/3**	2/2	4/4	**2/2**	1/2	1/1	0/2	1/2	**1/1**	2/2	1/2		
Grenache	IPSP	3/3	0/4		0/3	0/2								

*Abbreviations*: *SP* self-pollination, *EMS+ST* emasculation without sigma removal, *EMS-ST* emasculation with stigma removal, *EMS+OP* emasculation without covering, *stage I and II* E-L 15 and E-L 18, respectively, according to Eichhorn and Lorenz scheme [54]

^a^This category includes partial stigma damage (from 5 to 50%) for Sangiovese, Corinto Nero, Nebbiolo and Gamay at IPSP in 2014

**Fig. 8:**
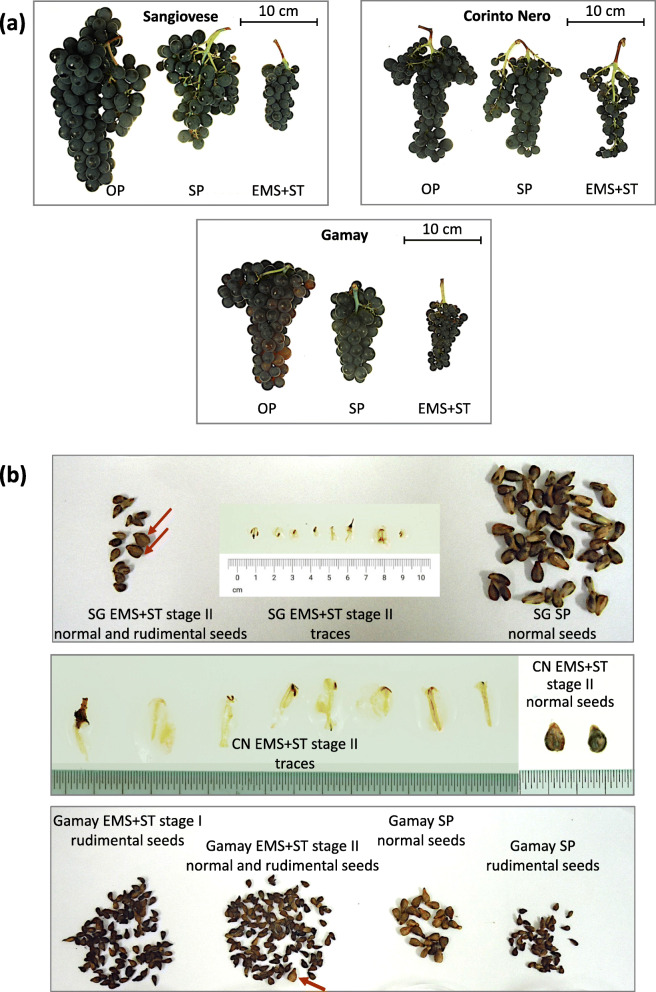
Clusters (**a**), seeds and traces (**b**) derived from open-pollination (OP), self-pollination (SP) and emasculation without stigma removal (EMS + ST). Other abbreviations: CN = Corinto Nero, SG = Sangiovese, stage I = stage E-L 15, stage II = stage E-L 18 of the modified Eichhorn-Lorenz scheme [54]. Red arrows indicate apparently normal seeds among several rudimental seeds

Sangiovese clusters derived from emasculated inflorescences showed only a few large berries (class A) with seeds (from 1.9 to 8.2% when pooling berries from all clusters). Most berries were significantly smaller (classes B and mainly C) compared to the control and contained traces of reproductive structures instead. These traces included very small remnants (ovule or seed traces) as well as notable rudimental seeds. Corinto Nero clusters derived from emasculated inflorescences resembled control bunches: very few large berries that harbored seeds were developed (from 0.4 to 7.6%), whilst the majority of berries were small (class C) and contained tiny traces (likely unfertilized ovules). Gamay clusters and berries formed after emasculation were smaller with respect to the control. Only a few berries (0.6% in 2015) showed normal seeds, whereas most berries accommodated rudimental seeds (Fig. 2c, Fig. 8 and Additional file 7: Figure S11).

All the seedlings derived from occasional normal seeds extracted from emasculated bunches, that are four plants from Gamay, three from Sangiovese and one putative from Nebbiolo, had a microsatellite profile that was fully compatible with self-pollination. Interestingly a Gamay seedling deriving from emasculation was homozygous for the SSR markers analyzed (Additional file 1: Table S8). Some of the seedlings had variegated leaves with green and albino sections.

#### Evaluation of female gamete (embryo sac) functionality

The four emasculated inflorescences of Corinto Nero that were manually pollinated with Nebbiolo pollen set fruit (Additional file 7: Figure S12). However, most berries were of medium or small size (97.3%) and did not contain seeds (95.5%); the few recovered seeds failed to germinate.

#### Exploration of potential causes of gamete non-functionality

In 2016, 629 seeds were extracted from the Corinto Nero seeded berries occasionally obtained by open-pollination. About 28% (against 95% in Sangiovese) were kept for sowing, as they were potentially viable (non-floating). The percentage of in vitro germination was similar in Sangiovese (54%) and Corinto Nero (51.5%) (Table 2). A total of 67 Corinto Nero seedlings were analyzed for ploidy level and genotyped at unlinked microsatellite loci (Additional file 1: Table S9). According to flow cytometry analysis, these plants had different ploidy levels. In particular, 42 plants were 4C (probable tetraploid), 14 were 3C (probable triploid), eight were 2C (probable diploid) and three were 6C (probable hexaploid). The Corinto Nero offsprings showed three different genotypes: 48 individuals (72%) displayed the same genotype as Sangiovese/Corinto Nero plants (Corinto Nero-like, type 1 according to [21]); 14 (21%) had the same genotype as Sangiovese/Corinto Nero plus additional exogenous alleles in several loci (Corinto Nero-like + exogenous alleles, type 2); five plants (7%) exhibited loss of Sangiovese/Corinto Nero heterozygosity in one or more microsatellite loci as well as additional exogenous alleles in several loci (Corinto Nero segregant + exogenous alleles, type 3). No plant had a profile consistent with being derived from regular self-fertilization (type 4).

Overlapping of ploidy and microsatellite data revealed that 42 out of 48 type 1 offspring were 4C, suggesting that they were generated by fertilization of a diploid Corinto Nero female gamete by a diploid Corinto Nero male gamete or, as an alternative, they derived from a tetraploid Corinto Nero egg cell. Of the six remaining Corinto Nero-like genotypes, two were 2C (probable apomixis), one was 3C (possible fertilization of a diploid Corinto Nero egg by a haploid Corinto Nero sperm nucleus or vice versa) and three were 6C (possible fusion of a diploid and a tetraploid gamete). Thirteen out of 14 type 2 plants were 3C, indicating the fertilization of a diploid egg cell by a haploid non-Corinto Nero sperm cell, while one was 2C, which needs to be better understood. Finally, all five type 3 plants were 2C, which is consistent with the fertilization of a haploid egg by a haploid non-Corinto Nero sperm cell. While no Corinto Nero self-crossed offspring plants were identified, the above genotypes suggest that only in a few cases (at most 6) regular Corinto Nero haploid female gametes might have been formed through meiotic reduction.

Pollen morphometric data, which were collected in view of the generally accepted correlation between pollen grain size and ploidy level, highlighted the great size variability of Corinto Nero pollen, due to heterogeneous and extreme values (15–36 μm, Fig. 7c) that are not usually observed in grape cultivars [55, 56]. About half of Corinto Nero pollen grains showed diameters lower than 22 μm and, similarly to Corinto Bianco pollen grains, they were on average smaller compared to those from other varieties, including Sangiovese. Moreover, several Corinto Nero pollen grains were collapsed and/or damaged.

In conclusion, our findings suggest that the seedless phenotype of Corinto Nero is driven by pollen and/or embryo sac defects, and a possible responsible mechanism is gamete non-reduction.

### Investigation of the molecular basis of the seedless phenotype

In order to identify genes possibly underlying the seedless phenotype of the investigated variants, we carried out two experiments. First, we tested all the accessions for the stenospermocarpy causal mutation in the gene *VvAGL11* to understand if it is required also in stenospermocarpic genetic backgrounds different from Sultanina. Then, we searched for single nucleotide polymorphisms between Sangiovese and Corinto Nero by exploiting the transcriptomic data produced by [52]. Validated variants were finally tested on additional clones and accessions of Sangiovese/Corinto Nero and on the other genotypes.

#### VvAGL11

Genotyping with the CAPS-26.88 marker confirmed that the Sultanina accession used in this study had the point variation (G > T) causing the stenospermocarpy-associated Arg197Leu substitution in the *VvAGL11* gene. All the other accessions were homozygous for the seeded allele (G/G), with the only exception of Aspirant. This accession was genotyped several times for the SNP position, corroborating the G/T genotype. Such polymorphism differentiated Aspirant from its seeded counterpart, Liseiret (data not shown).

#### Genes with validated SNPs between Sangiovese and Corinto Nero

A total of 71,557 SNPs and 37,121 INDELs satisfied the initial filtering criteria. From this list, it was required for any position to be considered a candidate SNP, to be present in at least two libraries and to be different between Corinto Nero and Sangiovese. This approach identified 1670 SNPs. When combined with variant effect prediction and functional gene annotation, 99 missense SNPs were selected for Sanger sequencing. Of these, five were confirmed to be true polymorphisms (Table 6 and Additional file 1: Table S10). All but one were retrieved in additional plants of Sangiovese (clones R10 and VCR4) and Corinto Nero (four accessions from Sicily). The only exception was the 4148 C > T variant on chromosome 6, which was uniquely found in the Corinto Nero accession from Calabria, the one deeply investigated in this study (data not shown).

**Table 6:** Missense single nucleotide polymorphisms between Sangiovese (SG) and Corinto Nero (CN) that were detected in the RNA-Seq dataset described by [52] and confirmed by Sanger sequencing. For each variant allele (highlighted in bold), somatic line specificity in comparison with the PN40024 (PN) reference genome, position, locus affected, locus functional annotation, genotype of each cultivar, and predicted effect in the protein sequence and function are shown.

Variant type	Variant position	Gene ID	Functional annotation	Genotype			Variant allele effect on protein sequence	Variant amino acid effect on protein function	PROVEAN score^**a**^
				PN	SG	CN			
SG specific	Chr2:2,837,618	VIT_02s0025g03330	H(+)-ATPase 4 AHA4	A:A	A:**G**	A:A	Y274C	Deleterious	-7.74
CN specific	Chr6:4,776,574	VIT_06s0004g03800	DNA-binding protein	C:C	C:C	C:**T**	T1383M	Neutral	-1.06
CN specific	Chr11:2,940,013	VIT_11s0016g03590	Transducing protein	A:A	A:A	A:**G**	I1114V	Neutral	0.48
CN specific	Chr11:5,363,589	VIT_11s0016g05820	CCR4-NOT transcription complex subunit 10	G:G	G:G	**A**:G	V317M	Neutral	-0.76
SG specific	Chr14: 23,114,120	VIT_14s0083g00910	Auxin-independent growth promoter	C:C	C:**T**	C:C	R536C	Deleterious	-2.71

^a^Variants with a PROVEAN score equal to or below -2.5 were considered "deleterious," variants with a score above -2.5 were considered "neutral"

The same sequences were obtained using either DNA isolated from root/berry pulp or skin tissues of Corinto Nero (data not shown).

## Discussion

This study was aimed at understanding the basis of some of the variation affecting seed and fruit development in grapevine. To this purpose, eight pairs of somatic variants with contrasting seed content were investigated.

### Phenotyping variant pairs

The members of each pair were phenotyped in the same vineyard and over multiple growing seasons in order to minimize the effect of environmental conditions and viticultural practices on their reproductive development [11, 57]. For example, micronutrient (in particular Zinc and Boron) deficiency might originate parthenocarpic fruit set [58, 59]. Moreover, a great degree of berry transcriptomic plasticity is documented for some genotypes like Sangiovese [60].

Firstly, seedless variants were compared to their seeded counterparts in terms of seed content, both by counting the number of seeded berries and by observing seeds and traces of reproductive structures at the stereomicroscope. This allowed us to confirm or attribute for the first time a seedlessness type to most variants: parthenocarpy to Corinto Nero, Moscato Bianco mutant, Termarina Rosa and Corinto Bianco, stenospermocarpy to Aspirant, Chasselas apyrène and Sultanina. In the case of Termarina Nera, further investigation is necessary. The complete absence of remains of unfertilized ovules in Moscato Bianco mutant suggests that its parthenocarpy is of the vegetative type [15]. Similarly to what observed in Korinthiaki/Black Corinth [19, 20] and in Corinto Bianco [21], the parthenocarpic phenotype of Corinto Nero and Moscato Bianco mutant proved to be not uniformly expressed, as revealed by the occasional appearance of individual berries with normal size and seeds in several clusters. Even if we never detected normally sized and seeded berries in Termarina Rosa, we cannot exclude that they may occur also in this parthenocarpic variant, as reported by [51].

Then, we investigated the effect of seed content on fruit set, berry size and bunch features. Consistent with the reported positive correlation between fruit set rate and number of seeded berries or seed number per berry [61, 62], fruit set rate (as assessed in 1 year, 2018) proved to be compromised in the examined seedless variants compared to their seeded counterparts, with the only exception of Aspirant (Fig. 1e and Additional file 1: Table S2). In addition to flower density (which is higher in all the seedless variants but Aspirant and Moscato Bianco mutant, Fig. 1d and Additional file 1: Table S2), putative factors affecting fruit set are flower fertility and pollination efficiency [63–65], a reduction of which may compromise seed set (as for Corinto Nero).

In the seedless lines compared to their equivalent seeded cultivars, we observed a clear predominance of lighter and smaller berries (Fig. 1g and Fig. 4), which resulted in lighter, smaller and generally looser bunches (Fig. 1h-j and Additional File 1: Table S3). Indeed, mean berry weight proved to be positively correlated with seed number and seed weight, in agreement with previous reports [61, 66–68]. The generally accepted explanation is that seed content influences berry growth (especially affecting cell division) through hormonal mechanisms, more seeds or larger seeds producing more hormones than fewer or smaller ones [2, 69]. In the seedless variants for which both diameters were measured, the decreased berry weight was associated to an evident spherical shape (Fig. 4). This could be due to pleiotropic effects on fruit size and shape. It is noteworthy that [37] documented a negative correlation between fertility index and berry traits, in particular berry shape index (length/diameter ratio).

### The occurrence of berry set after emasculation

Whilst unpollinated and unfertilized flowers usually abscise, Sangiovese, Corinto Nero and Gamay emasculated and bagged inflorescences were repeatedly observed to set fruit (Table 5). In all three genotypes, only a few normal-sized berries contained seeds, whereas the majority of berries were small and accommodated rudimental seeds or unfertilized ovules instead (Fig. 2c, Fig. 8a-b and Additional file 7: Figure S11). This phenomenon is not reported as a characteristic grapevine feature [70] and establishing the underlying biological mechanism is especially interesting.

Emasculation was performed when flowers were still closed; therefore, cross-pollination mediated by wind or by insects has to be excluded, as confirmed by the self-pollination compatible microsatellite profile of the few seedlings derived from germinated viable seeds (Additional file 1: Table S8). The segregation of SSR alleles, along with the paucity of fertile seeds, is also against the involvement of apomixis, which is asexual reproduction through seed [71].

Cleistogamy (self-pollination without calyptra fall) or bud-pollination (self-pollination taking place before the flower opens) might be possibly engaged. The occurrence of these phenomena has been hypothesized in some cultivars, while not appearing in others [72]. For example, [73] reported that at the time of opening, anthers in all flowers of Müller-Thurgau and Pinot Noir had already dehisced. About 16–18% of the flowers of Pinot Noir and 60–63% of Müller-Thurgau proved to be pollinated before opening and growth of pollen tubes had already started. [74] observed that about 2 weeks before anthesis Cabernet Sauvignon anther membranes were degraded and mature pollen grains had been released, while the cap was still attached to the flower. At this stage, an early seed structure had begun to develop. Given the assured seed set by cleistogamy and bud-pollination and the viability of Sangiovese (this work) and Gamay pollen [75], these methods of self-pollination triggered before emasculation might eventually have played a role in fruit set following emasculation, especially for the few normal-sized seeded berries. Nevertheless, we consider this hypothesis unlikely because at the time of flower emasculation anthers were still green and had not dehisced yet.

Likewise, we cannot exclude that, while castrating, some anthers bursted and allowed the pollen to escape, as already reported by [76, 77]. If some pollen by this time was already mature, it might have retained its vitality until the pistils became receptive, especially in flowers emasculated just before blooming.

In any case, we believe that the prevalent mechanism underlying berry formation (mainly small and seedless) after inflorescence emasculation, not only in the seedless genotype (Corinto Nero) but also in Sangiovese and Gamay, should have been parthenocarpy. This is also proved by the occurrence of inflorescences setting fruits after removal of both anthers and stigma during emasculation (Table 5). Indeed, grapevine has a characteristic facultative parthenocarpy, of both the vegetative (not requiring pollination) and the stimulative (requiring pollination) types, a phenomenon that intensifies when proper pollination is prevented by emasculation or by adverse environmental conditions [2, 57, 78, 79]. Previous reports of parthenocarpic fruits produced by emasculating and bagging the flower clusters are available for White Corinth, Black Monucca, Himrod seedless, Sultanina, Red Globe, Campbell Early and Muscat of Alexandria. In particular, this last variety was observed to produce some berries without seeds, some berries with empty seeds, some berries with seeds that had an endosperm and some berries with seeds that contained an embryo [2, 78, 80, 81]. This parthenocarpic potential might be an intrinsic property of grapevine (not restricted to specific genotypes) that becomes only expressed in the absence of fertilization upon certain conditions. In the case of emasculation, whether or not these special conditions exist, the successful outcome of this process might be considerably affected by the timing of emasculation (as shown for Sangiovese, Table 5). In the present study, it is noteworthy that the accessions setting fruit after emasculation (Sangiovese, Corinto Nero, and Gamay) are all early-flowering varieties. It is conceivable that in these genotypes developmental processes had progressed enough to result in ovary growth into fruit after removal of suppression signals from stamens or after perception of other signals in response to the damage of reproductive structures. Conversely, it is likely that in late-flowering cultivars (Grenache, Nebbiolo, Trebbiano Toscano, etc) emasculation was done too early in terms of reproductive organ development. Indeed, the stage of inflorescence development as determined by the E-L scale does not necessarily reflect the stages of development for the fertile organs (gametes), especially at key steps such as meiosis. In particular, the duration of reproductive organ development between meiosis and bloom is cultivar-dependent [82, 83]. An effect of developmental timing on fruit set is also supported by the observation that flowers that open first have less probability to abscise than the flowers that open later within the same cluster, because of polar auxin transport [84]. However, we cannot exclude that the individual genotype plays a role in this phenomenon, which could be enhanced in certain cultivars. For example, a study evaluating the reproductive performance of ten grapevine varieties [79] showed that Sangiovese is characterized by high bunch weight, high fruit set, high number of seeded and seedless berries, low proportion of live green ovaries relative to the total number of flowers, low coulure index (proportion of flowers that do not develop into either a berry or a live green ovary). Similarly, another study assessing the reproductive performance of 120 varieties [62] classified Sangiovese and Gamay into a group characterized by higher fruit set rates and lower coulure values, lower number of flowers and an intermediate number of seeded berries with respect to the other classes. The above features come out in favour of an intrinsic predisposition of these two cultivars to set fruit. Similarly to what we observed for Sangiovese, Corinto Nero and Gamay, some degree of background parthenocarpy following emasculation and coincident elimination of inhibitory signals from floral whorls surrounding the carpel was also seen in Arabidopsis ecotypes, several tomato lines and sweet pepper genotypes [85, 86]. Further experiments will be necessary to distinguish these hypotheses (grapevine intrinsic or genotype-dependent property).

### Understanding the basis of the variation in seed development

The reasons of seedlessness could be related to abnormalities in ovule formation before flowering, low level of pollen fertility, insufficient pollination and fertilization at flowering, embryo/endosperm abortion after fertilization.

In tomato, Arabidopsis and *Capsicum annuum* [86–90] parthenocarpy has been found associated with alterations in early ovule development (defective integument growth and irregular meiosis reducing the production of viable female gametes). A connection between parthenocarpy and ovule defects exists also in grapevine; ovule development anomalies can occur before megasporogenesis (in White and Red Corinth according to [20]), at the end of megasporogenesis (in Corinto Bianco to [21]) or during megagametogenesis (in Black Corinth to [20]). In the present work, the majority of berries derived from the cross Corinto Nero x Nebbiolo or from open-pollination of Corinto Nero had no seeds. This indicates that the availability of viable pollen (from Nebbiolo or other cultivars in open-pollination) is not sufficient to promote normal seed development in Corinto Nero and that female defects contribute to impeding this process. We hypothesize that, at the time of anthesis, Corinto Nero embryo sacs are missing or in various stages of degeneration, rarely able to function in fertilization. In fact, the ploidy level of Corinto Nero seedlings evidenced anomalies during meiosis in megasporogenesis. Therefore, Corinto Nero seedlessness is likely due to the lack of functional female gametes coupled with an alternative fertilization-independent process of fruit development.

Parthenocarpy has been additionally associated to male sterility in mutants and transgenic lines of tomato and apple, largely involving genes that control floral organ identity and development [87, 91–96]. Consistently, a relationship between seed set and pollen viability or germination has been documented in grapevine, with low pollen fertility resulting in a low level of seed setting, due to an increased probability of pollination failure [21, 64, 97]. The in vitro tests performed in the present study revealed that Sangiovese pollen is viable and able to germinate, even if at lowest levels in the range of variation reported for grapevine cultivars ([61, 65, 75] and references therein, [98]). Oppositely, its seedless variant Corinto Nero showed negligible pollen viability and germination, as the parthenocarpic Corinto Bianco (Fig. 7a-b and [21]). The very low presence of viable pollen grains in Corinto Nero might explain the only occasional formation of seeded berries after self-fertilization in case of rarely available functional ovules (Additional file 1: Table S7). The non-functionality of Corinto Nero pollen was also supported by the in vivo pollination experiments, that are comparison of self- and open-pollination (Additional file 1: Table S7) and cross-pollination of Nebbiolo and Trebbiano Toscano (Table 4). We exclude that the poor berry set following pollination of these two highly productive cultivars with Corinto Nero pollen was determined by a time-shift in the reproductive development of donor and recipients, because Nebbiolo and Trebbiano Toscano have different flowering times.

Similarly to what found for Corinto Nero and Corinto Bianco, a reduced flower fertility can be hypothesized to be also at the basis of Moscato Bianco mutant parthenocarpy. This accession shows typical“star” flowers, with petals freely opening from the top of the calyptra instead of abscising from the base and being subsequently shed fused together as a “cap”. Stamens are short and anthers remain stuck to the calyptra. Such conformation was earlier observed in numerous varieties and it was associated to male sterility, aberrant ovules with incomplete integuments (equated with ovules from White and Red Corinth described by [20]), poor fruit set and parthenocarpic berry development [99, 100]. Unfortunately, we do not have enough data to draw any conclusion or hypothesis about Termarina Rosa. In all the investigated parthenocarpic variants where both female and male gametes are affected, it is likely that a single mutation could be responsible for both effects.

As concerns stenospermocarpy, seed abortion in Sultanina and related seedless table grape varieties has been previously attributed to the Arg197Leu substitution in VvAGL11. A working model has been proposed in which the Arg197Leu mutation disrupts the function of multimeric complexes containing VvAGL11 proteins. In turn, this prevents proper seed coat differentiation and finally leads to endosperm degeneration and embryo development arrest [32, 101]. Given the observation of a stenospermocarpic phenotype for Aspirant and the detection of the same mutation in *VvAGL11*, we hypothesize that the same events taking place in Sultanina lead to Aspirant seedlessness. Oppositely, we envisage another source of stenospermocarpy for Chasselas apyrène that does not carry the Arg197Leu mutation. As in the case of Sultanina [61] and of another stenospermocarpic variety (Parvana [98]), we exclude that Chasselas apyrène seedlessness is related to pollen non-functionality (Fig. 7).

### Potential causes of gamete non-functionality

Non-functional gametes may be the result of failure at different points in their development. In particular, irregularities may take place during sporogenesis, during the development of surrounding structures like tapetum and nucellus or during the final steps of gametogenesis.

Meiosis omission or abortion involving both micro and macrosporogenesis is a likely cause of Corinto Nero sterility and impeded seed formation, as reported for Corinto Bianco [21] and to a lesser extent also for other varieties [97, 102]. Indeed, the genetic analyses of Corinto Nero seedlings (Additional file 1: Table S9) revealed that Corinto Nero infrequent functional male and female gametes are mostly unreduced gametes (as inferred from 62 out of 67 seedlings), and the major part of unreduced gametes are diploid (originating at least 58 seedlings). These diplogametes might derive from apomeiosis (suppressed or imperfect meiosis), which is the first step of gametophytic apomixis [103]. The presence of two diploid Corinto Nero-like seedlings (type 1) supports, in facts, the involvement of apomixis in these two cases. Although they are typically much more frequent events among apomicts, both the formation of unreduced gametes and the parthenogenetic development of unfertilized egg cells are widely recorded phenomena in sexual species [104]. It is conceivable that the type of apomeiosis occurring in female gametes here is diplospory. Although diplogametes may derive from a variety of different meiotic abnormalities, they all result from one of two basic processes depending on the mode of nuclear restitution: First Division Restitution (FDR) and Second Division Restitution (SDR), which occur during abnormal development of the first and the second meiotic divisions, respectively. FDR produces gametes containing non-sister chromatids, which retain the whole or a large part of parental heterozygosity [105, 106]. SDR gametes, instead, possess sister chromatids [107]. Therefore, to further elucidate the ontogeny of Corinto Nero female diplogametes we focused on the genetic make-up of triploid seedlings at microsatellite loci that are heterozygous in Corinto Nero, as suggested by [21]. Segregation of Corinto Nero alleles was never observed in the triploid seedlings obtained in the present work and the only type 3 Corinto Nero offsprings (segregant + exogenous alleles) were diploid. This result is consistent with the occurrence of FDR, but it does not exclude the involvement of apospory apomeiosis. Cytohistological studies would be required to determine the origin of the diploid precursor cell. In addition, since in tetraploid and hexaploid Corinto Nero offsprings potential losses of heterozygosity produced by meiotic segregation events are masked by chromosome duplication, we cannot rule out that additional 2n gamete-inducing mechanisms (like SDR) may occur, as observed in other plants [108]. As it has been hypothesized for Corinto Bianco [21], Corinto Nero might be a meiotic mutant with a recessive homozygous mutation or, more probably in a somatic variant, a dominant heterozygous mutation [109].

The variability in Corinto Nero gametophytic ploidy level is well reflected in the wide variability in pollen size (Fig. 7c), in agreement with the generally accepted correlation between pollen grain size and ploidy level [105, 107]. In particular, the bigger pollen grains might correspond to viable diploid pollen grains, as proposed in the case of Corinto Bianco [75]. Based on the above discussion, it is also tempting to speculate that the greater size of Corinto Nero occasional seeds compared to those of all other accessions that were inspected at veraison (Additional file 1: Table S5) is the result of the involvement of unreduced gametes in fertilization.

Other reasons of pollen non-functionality may be shape abnormalities and lack of furrows or germinative pores, which implicate a morphological sterility [110, 111]. In the present work, several Corinto Nero pollen grains were found to be collapsed and it is conceivable that additional structural aberrations might be responsible for negligible viability/germination of Corinto Nero pollen grains. However, a more focused microscopic investigation would be necessary to prove it.

### The genes possibly underlying the seedless phenotype

#### VvAGL11

Based on the genotype at CAPS-26.88 marker, we suggest that Aspirant stenospermocarpy is linked to the Arg197Leu missense substitution in *VvAGL11*, similarly to what was found for Sultanina by [32]. On one side, this could indicate that a very specific mutation is required for stenospermocarpy based on *VvAGL11* gene. On the other side, the seedless variant of Iordan (Gouais/Liseiret offspring) was homozygous for the seeded allele. This could mean that other mutations in the same gene are potentially involved (since they have not been discarded in the present work) or another source of seedlessness exists, as conceivable for Chasselas apyrène as well. Different seedlessness mechanisms (not involving *VvAGL11*) are also expected in the parthenocarpic variants analyzed here.

#### Genes with validated SNPs between Sangiovese and Corinto Nero

In the last years, different molecular mechanisms responsible for somatic variation have been identified, including point mutations, insertions/deletions of transposable elements and chromosomal rearrangements (for a review see [109]). Based on this knowledge, we took advantage of the transcriptomic experiment done by [52] to perform a preliminary investigation of single nucleotide polymorphisms between Sangiovese and Corinto Nero. Five SNPs were validated, which have a potential involvement in intra-varietal phenotypic variation. Even in the absence of any functional role, these polymorphisms might be useful to discriminate Sangiovese and Corinto Nero.

Considering that both Sangiovese and Pinot Noir are seeded varieties, the most interesting genes (with possible causal SNPs) are those showing a PN40024-like genotype in Sangiovese and a variant nucleotide in Corinto Nero, that are VIT_06s0004g03800 (4148 C > T), VIT_11s0016g03590 (3340 A > G) and VIT_11s0016g05820 (949 G > A) (Additional file 1: Table S10). The phenotypic effect might derive from gain-of-function mutations or from loss-of-function mutations resulting in haploinsufficiency [36]. However, these variants were predicted to have a neutral effect on protein function according to PROVEAN score (Table 6).

VIT_06s0004g03800 codes for a nuclear factor related to kappa-b-binding protein. The product of the orthologue Arabidopsis gene is a component of INO80 chromatin-remodelling complex. The roles of SWR1(SWi2/snf2-Related 1)/INO80-complex in nuclear activities are quite diverse ranging from double-strand breaks repair to regulation of gene expression. Interestingly, some core SWR1/INO80-c subunits have been shown to act in reproductive development, e.g. female meiosis, in Arabidopsis [112]. The 4148 C > T SNP determines a Thr1383Met change, which corresponds to a change in aminoacidic polarity.

VIT_11s0016g03590 codes for a transducing protein; the variant aminoacid falls within a conserved WD40 repeat. Repeated WD40 motifs are known to act as a site for protein-protein or protein-DNA interaction, and proteins containing WD40 repeats serve as platforms for the assembly of protein complexes or mediators of transient interplay among other proteins. These proteins are implicated in a variety of functions ranging from signal transduction and transcription regulation to cell cycle control and apoptosis [113]. Out of the five genes containing SNPs, VIT_11s0016g03590 is the only one with a differential expression between Sangiovese and Corinto Nero, which is a significant up-regulation from E-L 15 to E-L 27 only in the seedless clone [52].

The product of VIT_11s0016g05820 is a component of the CCR4-NOT complex, which is one of the major cellular mRNA deadenylases and is linked to various processes including mRNA degradation, miRNA-mediated repression, translational repression and general transcription regulation [114]. Interestingly NOT1, the scaffold protein of the CCR4-NOT complex, has been recently established as an important player during male and female gametophyte development in Arabidopsis, with its disruption showing abnormal seed set [115, 116]. Surprisingly, the variant 949 G > A was also found in a homozygous state in the stenospermocarpic Chasselas apyrène (Additional file 1: Table S10), which makes even more intriguing to understand if it plays a role in the seedless phenotype(s).

The two Sangiovese-specific validated SNPs are in the genes VIT_02s0025g03330 and VIT_14s0083g00910. Notably, these variants were predicted to have a deleterious effect on protein function according to PROVEAN score (Table 6).

VIT_02s0025g03330 codes for an autoinhibited H^+^ ATPase (AHA) and the variant aminoacid is in a conserved E1-E2_ATPase domain. Some AHA isoforms have been suggested to play a major role in male gametophyte formation and function [117], in particular in microspore development, e.g. [118], and in pollen tube growth ([119] and references herein). Other members of the AHA family have been shown to be involved in seed coat endothelium development and in embryo viability [120]. This gene falls within the confidence interval of QTLs for cluster weight and compactness, as well as rachis and shoulder length [121].

The product of VIT_14s0083g00910 is a fucosyltransferase with a potential role in pollen tube growth [122]. This gene is comprised in the confidence interval of QTLs for seed weight [28, 32], number of berries per cluster [123, 124], rachis length [125], number of nodes of the central cluster axis [124] and flowering time [126].

At four SNP positions, all the five analyzed clones of Corinto Nero shared the same allele, which hints at a common origin and propagation history. The presence of the 4148 C > T mutation in a single Corinto Nero clone (the one from Calabria deeply investigated here) suggests instead that this mutation is relatively recent (data not shown).

Based on the analysis of DNA extracted from different organs (layer-specific approach), a chimerical nature of the clones for the identified mutations could be excluded. This result, which contrasts with the quite common somatic chimerism reported in grapevine clones ([36, 127] and references therein), can be explained by cell layer rearrangements leading to homogenization of the plant genotype [128, 129].

## Conclusions

The present study shows that genetic diversity preserved in grape germplasm collections may be crucial for investigating the regulation of target traits. Here, independent seedless variants were characterized at the molecular and phenotypic level. Multi-year observations on seed and fruit set deriving from different pollination treatments allowed us to attribute each genotype a biological mechanism leading to seedlessness, between parthenocarpy and stenospermocarpy. The missense substitution in *VvAGL11* that is responsible for seed abortion in Sultanina-derived seedless varieties was not detected in the seedless variants evaluated in this work, with the only exception of an apparently independent Gouais Blanc mutant. For the Corinto Nero (Sangiovese seedless variant) case study, specific defects were identified in micro- and macro-gametophytes, which act in concert to promote parthenocarpy. Moreover, evidence was found in support of the intrinsic predisposition of Sangiovese and Corinto Nero to set fruit even in the absence of fertilization. Based on RNA-Seq sequence data, some hypotheses were developed on genetic functions that might be altered in Corinto Nero.

## Methods

### Plant material

Seven seeded *Vitis vinifera* varieties and their corresponding seedless somatic variants were selected for genetic and phenotypic characterization (Table 1). The plants are currently grown in two grape germplasm collections in northern Italy: the Grinzane Cavour collection (http://www.ipsp.cnr.it/grape-collection/?lang=en), which is located in the province of Cuneo and is maintained by CNR-IPSP (National Research Council of Italy-Institute for Sustainable Plant Protection, Torino); the FEM collection (https://www.fmach.it/eng/Farm/Crops/Corporate-bodies/Giaroni-San-Dona), which is situated in the province of Trento (experimental field “Giaroni” in San Michele all’Adige) and is managed by FEM (Fondazione Edmund Mach). The most investigated accessions in this study, Sangiovese and Corinto Nero, have been propagated and planted in two additional locations that are an experimental field in Grugliasco (Torino) and another in San Michele all’Adige. In all the vineyards, vines are grown in vertical trellis and Guyot pruned.

Corinto Nero was initially identified as a seedless somatic variant of Sangiovese collected in the region of Calabria (southern Italy, precisely in Scalea, Cosenza province) and introduced in the Librandi winery collection, as described by [41]. Another Sangiovese seedless mutant was found in Emilia Romagna (Sesso, Reggio Emilia), under the name of Termarina Nera. The main reference seeded Sangiovese was the clone R24.

The seedless variant of Moscato Bianco was discovered in a Moscato Bianco commercial vineyard in the region of Piemonte (precisely Alba, Cuneo). Aspirant-false, the seedless variant of Gouais Blanc/Heunisch Weiss, was kindly provided by the JKI Geilweilerhof, Germany.

Termarone and its seedless variant Termarina Rosa were identified by microsatellite analysis and introduced in collection from the Italian region of Emilia Romagna.

The somatic variants of the cultivars grown in the FEM collection were found by investigating the seed phenotype (number and type of seeds from 25 randomly sampled berries in 2011 and 2012) within groups of accessions with identical profile at 22 microsatellite loci and a name possibly referring to seedlessness [130]. Three pairs of somatic variants were at that time identified: Chasselas Blanc and Chasselas apyrène, Dastatchine-false (Sultanine Monococco) and Sultanina, Pedro Ximenez and Corinto Bianco. A fourth pair was discovered that included a Sori-false accession and Corinthe Noir (the Greek Korinthiaki). However, Sori-false was then excluded from phenotypic characterization as the putative seeded form of Corinthe Noir because in the following seasons, when all bunches were examined, most of the berries were small and seedless. Both accessions proved to be subject to reiterative berry shriveling. When phenotypic data could be collected, Corinthe Noir was kept as a reference for parthenocarpy.

Additional cultivars, clones or accessions of the above cited and of other varieties were analyzed for specific goals, as detailed along the manuscript. They include: Sangiovese clones R10 and VCR 4; four accessions of Corinto Nero from Sicily (Aeolian Islands); Chasselas Rose, a seeded berry color variant of Chasselas Blanc; Sultanina Rosa, a berry color variant of seedless Sultanina; Iordan, a Gouais/Liseiret offspring and its variant Iordan seedless; lastly, Gamay, Grenache, Nebbiolo and Trebbiano Toscano for pollination treatments.

For simplicity, we often drop the term “false” for accessions wrongly labelled.

### Genotyping variant pairs

For SSR and SNP genotyping, young leaves were gathered from all the accessions reported in Table 1. Total genomic DNA was extracted according to [130].

Sangiovese and Corinto Nero had been previously genotyped with 58 SSRs spread across the nineteen chromosomes of the grapevine genome [52]. The remaining accessions were analyzed with 32 out of these 58 markers (Additional file 1: Table S1).

In addition, each accession was genotyped with the commercial GrapeReSeq_Illumina_20K_SNP_chip [131] containing 18,071 SNPs. SNP genotypes were scored as described in [132]. An in house Perl script was used to carry out pairwise comparison of the filtered genotype positions for each pair of seeded and seedless accessions.

Potential polymorphisms between somatic variants were validated through PCR amplification and Sanger sequencing. In a few cases, a subset of putative SNPs was selected based on variant effect prediction (with the SNPeff v3.6c program by [133]) and on functional gene annotation. Primers were designed according to the 12X.2 version of the reference genome sequence using Primer3Plus [134]. PCR products were purified using Eurosap PCR Enzymatic clean-up kit (Euroclone S.p.A, Pero MI, Italy) and then sequenced by capillary electrophoresis with the same primers as in PCR. Chromatograms were aligned with MEGA6 software [135] and visually inspected with BioEdit v7.2.0 [136].

### Phenotyping variant pairs

The accessions reported in Table 1 (with their respective geographic locations) were phenotyped for flower and fruit traits upon open-pollination in one or more seasons. Developmental stages were established according to the modified Eichhorn-Lorenz scheme [54].

#### Flower number and fruit set rate

In 2018, fruit set rate was evaluated in both locations as the ratio of berries over flowers per bunch, which is the only valid method recognized by [62]. The number of flowers per inflorescence was assessed at stage E-L 17 (12 leaves separated; inflorescence well developed; single flowers separated) by using VitisFlower mobile application according to the developers’ specifications [137, 138]. As a preliminary step, the app reliability was tested by comparing in the lab the estimated flower number with the manual count for six Chasselas Rose inflorescences of different size (R^2^ = 0.90). For most accessions, five to ten inflorescences were then chosen from different plants and different positions within the plant, in order to minimize potential effects of branching level and inflorescence position along the shoot onto flower number [11, 139]. Three photographs per inflorescence were taken (from different angles) and a mean value was calculated. The number of berries set per bunch was manually counted at harvest (E-L 38) in the lab. For the plants of the FEM collection, berries were also manually counted in the field at stage E-L 31 (berries pea-size) by marking each berry with a permanent pen. Live green ovaries were not included in the counts, as they do not fit the definition of berry [140].

#### Bunch, berry and seed features

Bunch, berry and seed traits were evaluated on clusters collected at technological maturity (stage E-L 38) in one (2018 for Chasselas apyrène/Chasselas Rose, Sultanina/Dastatchine and Corinthe Noir) or more seasons (up to four for Corinto Nero/Sangiovese: 2013, 2016–2018; two for all the other accessions: 2017 and 2018).

Bunch features included the following OIV descriptors: length (OIV202), width (OIV203), mean cluster density (OIV204), as well as bunch weight, length/width ratio and berry number. Clusters were weighted with a precision balance. Bunch length and width were measured with a ruler. The number of berries per bunch was manually counted.

Berry traits included berry size, mean berry weight and percentage of seeded berries. Berries of each bunch were classified into three size categories: A or large (berry width > 15 mm), B or medium (12 mm ≤ berry width ≤ 15 mm) and C or small (berry width < 12 mm). Berry width (OIV221) was measured with a digital caliper applied to pictures (at IPSP in 2017 and 2018) or with an ad-hoc aluminum sizer card from 9 to 20 mm in 1 mm steps (at FEM in 2018). At IPSP, berry length (OIV 220) was additionally surveyed. Pools of berries of the same size class were weighted with a precision balance and an average berry weight was obtained for each cluster. The percentage of seeded berries per cluster was calculated after opening the berries with a blade and visually inspecting the presence of normally developed seeds.

Seed traits included mean seed number per seeded berry and mean seed weight. Normally developed seeds were extracted from berries of the same size category and manually counted. Fresh seed weight was measured with a precision balance after seed cleaning and drying at room temperature. An average count and weight were obtained for each cluster.

#### Inspection of seeds and traces of reproductive structures at veraison

In 2019, a pool of berries from different parts of different bunches was randomly collected at veraison for each genotype, except for Chasselas Rose, Pedro Ximenez and Corinto Bianco. Ten berries per size category (small and large, when available) per genotype were randomly chosen for inspection at the stereomicroscope. Traces (of ovules or seeds) and well-developed seeds were extracted from each berry and separately counted. The potential vitality of the well-developed seeds was tested by a floatation test in water: the sinking seeds were considered as likely viable. Traces and seeds were successively dissected for observation of their structures. A digital camera (AxioCam ERc 5 s, ZEISS) was attached to the stereomicroscope (Stemi 2000-CS, ZEISS) and simultaneously connected to a computer. AxioVision Rel. 4.8 software (ZEISS) was used to observe the samples in “live” mode and to get digital images. The size range of the analyzed berries, as well as the length and width of traces and seeds, were digitally measured from the pictures.

In addition, for Sangiovese and its seedless variant Corinto Nero, pistils from different inflorescences or from a single inflorescence with flowers at different phenological stages were collected on the same day (19/06/2019). Afterwards, four intermediate stages between flowering (stage 1) and berries pepper-corn size (stage 6) were sampled. For each genotype, one pistil per stage was selected for successive dissection, extraction and examination at the stereomicroscope of the ovules/seed traces. Their length and width were measured using the software cited above.

#### Statistical analysis of phenotypic data

Statistical tests were performed using the software PAST v3.14 [141]. Both parametric (T-student and Welch in case of unequal variance) and non-parametric (Mann-Whitney and Kolmogorov-Smirnov) tests were performed to detect significant differences between somatic variants or stages for berry count. Significant differences among different genotypes were additionally tested by using the Kruskal-Wallis test (with the Dunn’s post-hoc test and Bonferroni adjustment). A significance level of *P* < 0.05 was set in all cases. Pairwise correlations between traits were assessed with the Spearman’s rs test and considered for significance at the 0.05 level.

### Understanding the basis of the variation in seed development

#### Evaluation of sanitary status

In 2011 and 2012, woody material from vines was tested for the presence of the most harmful and spread grapevine viruses by applying ELISA (enzyme-linked immunosorbent assay) test and PCR as described in [142, 143]. Virus status of the investigated accessions is reported in Additional file 1: Table S11. It is worth specifying the mother plants from where the single vines planted in collection were propagated underwent tests for harmful virus load. When grafted, also the certified rootstocks used were controlled for being free from harmful viruses. Being the plants not older than 4–6 years, it is plausible all the investigated vines maintained the same virome profiles. Moreover, the collection vineyards are routinely monitored for potential vector occurrence.

#### Evaluation of male gamete (pollen) functionality

##### Pollen viability and germination

Pollen viability and germination were tested in vitro on Sangiovese/Corinto Nero and the three variant pairs Chasselas/Chasselas apyrène, Dastatchine/Sultanina, Pedro Ximenez/Corinto Bianco, as well as on Corinthe Noir cv. Inflorescences were collected at stage E-L 23 (50% caps off) of the modified Eichhorn-Lorenz scheme [54]. No selection was done for the inflorescence and shoot position, as pollen viability has been shown to be highly uniform within the same genotype [75]. Pollen viability and germination were analyzed over three seasons (2014, 2017 and 2018). For each accession, a pooled sample composed of inflorescences from different plants was tested.

Viability: The pollen viability of freshly harvested inflorescences was determined using the 1% TTC (2,3,5-triphenyl tetrazolium chloride) test [144]. TTC, normally colorless, in the presence of dehydrogenases (viable pollen) turns into insoluble formazan and appears red. For each sample, three or four technical replicates were performed, spreading pollen grains on different glass slides. After incubation in the dark at 37 °C for 1 h, pollen viability was evaluated under a microscope (Leitz Diaplan): pollen grains were considered viable if they turned red, non-viable if yellowish or unstained. Viable and non-viable pollen grains were counted in random samples of about 300 grains per slide.

Germination: In order to ensure pollen shedding from anther sacs and separation from other flower parts, inflorescences were sieved. Spontaneously released pollen grains were collected in a Petri dish and a germination medium (20% sucrose, 100 mg/L boric acid, 300 mg/L calcium nitrate) was added [145]. After 24 h of incubation at 25 °C [146], three slides were prepared for each sample and examined under a microscope (Leitz Diaplan) using the continuous sweep method and random sweep selection. The pollen grains were considered germinated when the length of the pollen tube was at least the double of the granule diameter. At least one-hundred pollen grains per slide were observed.

##### Pollination treatments

The following pollination treatments were performed:

A) Self- vs open-pollination: Seed and fruit set were evaluated in self- and open-pollination conditions (SP and OP, respectively) in most seeded/seedless pairs. The only exceptions were Termarina Rosa, Dastatchine and Corinto Bianco due to too few or dried inflorescences in 2018. For the self-pollination group, inflorescences were enclosed within paper bags before anthesis to avoid cross-pollination and were allowed to bloom and self-pollinate. One week after berry set, the covered clusters were exposed to full sun throughout fruit development and maturation (the same holds for B and C).
B) Pollination of Nebbiolo/Trebbiano Toscano with Corinto Nero pollen: Pre-capfall inflorescences of Nebbiolo and Trebbiano Toscano (cv. early and late flowering respectively, both fully fertile and seeded) were manually decapped, emasculated using pliers with fine tips, hand-pollinated with Corinto Nero pollen, and covered with paper bags. The experiment was repeated in different seasons (2012–2014) and locations (IPSP and FEM). Self-pollinated clusters (inflorescences enclosed in bags before calyptra shed) represented positive controls. Seed and fruit set were evaluated in both pollination conditions.
C) Emasculation of some pairs and additional varieties: Pre-capfall inflorescences of Sangiovese/Corinto Nero, Gouais Blanc, Chasselas/Chasselas apyrène, Pedro Ximenez/Corinto Bianco and additional genotypes (Nebbiolo, Trebbiano Toscano, Gamay, and Grenache) were manually decapped, emasculated using forceps with fine tips and covered with paper bags. The aim was to check the eventual berry set and development excluding any pollen role. This experiment was repeated in different seasons, locations and at different developmental stages. The earliest stage (stage I) corresponded to stage E-L 15, the latest one (stage II) to stage E-L 18. In some trials stigma removal was additionally performed. Undecapped self-pollinated (covered) inflorescences were used as control. Seed and fruit set were evaluated in both pollination conditions. Occasional normal seeds formed upon emasculation were placed in pots for germination. Derived seedlings were genotyped at 18 microsatellite loci to clarify their origin.

#### Evaluation of female gamete (embryo sac) functionality

In 2013, four inflorescences of Corinto Nero were emasculated and cross-pollinated with viable pollen of Nebbiolo with the procedure described above. Seed and fruit traits were evaluated at harvest.

#### Exploration of potential causes of gamete non-functionality: defects in sporogenesis

In 2016, Corinto Nero and Sangiovese seeded berries, obtained upon open-pollination conditions, were collected. Seeds were extracted from berries and stored at 4 °C for 2 months in order to overcome dormancy. Seed germinability was then evaluated for both accessions. In vitro embryo rescue was performed according to the protocol described by [21]. Young leaves were sampled from the obtained seedlings and they were divided into two batches. The first batch was used for genotyping at ten unlinked microsatellite loci (fifteen in some dubious cases). Leaves from the second batch were sent to Plant Cytometry (https://plantcytometry.com/) for ploidy level determination by flow cytometry. The ploidy level of each plant was recorded as an index relative to plants of the same species with a known ploidy level (2C), that are Corinto Nero, Sangiovese and Cabernet Sauvignon (leaves were collected from woody cuttings kept in pots with water).

In parallel, pollen grain morphology was recorded in Sangiovese/Corinto Nero (in 2014, 2016 and 2017) and in other three variant pairs (in one or two seasons, 2017 and 2018) to verify possible different size of pollen grains linked to different ploidy level. Polar and equatorial axes of 50 randomly taken pollen grains were measured for each genotype in each season by examination at light microscope using an ocular micrometer.

### Investigation of the molecular basis of the seedless phenotype

Candidate genes for the seedless phenotype were identified/analyzed in one or more variant pairs:

#### VvAGL11

All the accessions under study were genotyped with the CAPS-26.88 marker by using the primers reported in [32] for both PCR amplification and Sanger sequencing.

#### Genes with validated SNPs between Sangiovese and Corinto Nero

A preliminary search for single nucleotide polymorphisms (SNPs) between Sangiovese (clone R24) and Corinto Nero (from Calabria) was addressed by a two-step process. To this purpose, we took advantage of the RNA-Seq alignments used by [52] for differential expression analysis in the pairwise comparison of developmental stages in the two lines (six libraries in total, which correspond to three stages and two genotypes). In the first step, polymorphisms were sought between Sangiovese and/or Corinto Nero and the 12X.0 version of the grapevine reference genome. Variants were called with Samtools v0.1.17 [147]. An initial filtering was done with VCFtools v4.1 [148] using a window of 10 bp, a minimum read depth of five and a minimum quality of 10. Then, to identify differential single nucleotide variants between Corinto Nero and Sangiovese with a potential impact on the seed phenotype, the following approach was adopted:

A) Through VCF filtering, it was required that the alternative base was supported by at least 3 reads and the frequency of the alternative alleles was ≥0.75 calculated on the total number of read pairs aligned on the region;

B) An ad hoc Perl script was written to take consensus positions that pass the filtering criteria in at least two libraries (that correspond to two developmental stages and can be considered as replicates) of Sangiovese and Corinto Nero, respectively;

C) Putative mutations from B were annotated on *Vitis vinifera* V1 gene predictions by using the Variant Effect Predictor SNPeff v3.6c program [133];

D) An ad hoc Perl script was used to carry out a pairwise comparison between Sangiovese and Corinto Nero for all putative SNPs annotated as non-synonymous;

E) Ninety-nine putative SNP positions that are different in the two clones from D were further chosen for validation. This set includes all the non-synonymous SNPs supported by three libraries and a selection (based on gene function) of non-synonymous SNPs supported by two libraries out of three (due to missing or incoherent genotype from one library).

To validate the selected SNPs, PCR amplification and Sanger sequencing were initially performed on genomic DNA from young leaves of the two clones and of Pinot Noir (as a reference) by following the approach described in the section “Genotyping variant pairs”. Primer sequences are available in Additional file 1: Table S10. Individual inferred genotypes from RNA-Seq were checked for concordance with Sanger method.

For validated SNPs, predicted impact value on protein function was estimated with PROVEAN application [149]. The CD-Search tool available on the NCBI portal [150] was used to check whether those mutations affect conserved sites or domains.

Validated variants were then tested on additional clones and accessions of Sangiovese/Corinto Nero. Chimerism was also investigated by comparing the Corinto Nero genetic make-up in genomic DNA extracted from leaf/berry skin (L1 + L2-derived tissues) and in genomic DNA isolated from berry flesh/adventitious roots (L2-derived tissues) [151].

Finally, validated variants between Sangiovese/Corinto Nero were analyzed in the other wild-type/variant pairs and in Corinthe Noir. By using the tool “Sanger data analysis” of Unipro UGENE v1.32 [152] with default settings for quality filtering, amplicons were aligned against *Vitis vinifera* V1 gene predictions containing each SNP.

## Supplementary Information

**Additional file 1: Table S1.** Genotyping of variant pairs with SSR markers. **Table S2.** Statistical analysis of bunch, berry and seed traits upon open-pollination. **Table S3.** Bunch compactness according to the OIV 204 descriptor. **Table S4.** Spearman correlation between bunch compactness and other traits evaluated in this work. **Table S5.** Statistical analysis of length, width and length/width ratio of the apparently normal seeds extracted from berries at veraison. **Table S6.** Statistical analysis of length, width and length/width ratio of the ovule/seed traces extracted from berries at veraison. **Table S7.** Statistical analysis of seed and fruit set in open- and self-pollination conditions. **Table S8.** Microsatellite profile of Sangiovese, Gamay and Nebbiolo seedlings derived from self-pollination and emasculation with inflorescence bagging. **Table S9.** Corinto Nero offspring analyzed for ploidy level and microsatellite genotyping. **Table S10.** Single nucleotide polymorphisms and insertions/deletions identified in the five amplicons containing the RNA-Seq SNPs that distinguish Sangiovese and Corinto Nero. **Table S11.** Results of the tests for the presence of viruses.

**Additional file 2: Figure S1.** Trait stability in multiple seasons and locations upon open-pollination.

**Additional file 3: Figure S2.** Relationship between berry size and presence of normal seeds.

**Additional file 4: Figure S3.** Percentage distribution of Sangiovese and Corinto Nero berries according to seed content in two pollination conditions.

**Additional file 5: **Inspection of traces and seeds extracted from berries at veraison for the seedless accessions Aspirant (**Figure S4**), Chasselas apyrène (**Figure S5**), Corinto Nero (**Figure S6**), Termarina Rosa and Moscato Bianco mutant (**Figure S7**), Corinthe Noir and Sultanina (**Figure S8**), and for the seeded cultivars Liseiret, Moscato Bianco, Termarone and Sangiovese (**Figure S9**).

**Additional file 6: Figure S10.** Sangiovese and Corinto Nero pistils at six phenological stages, with details of ovules/seed traces for which length and width were measured.

**Additional file 7: Figure S11.** Clusters of Sangiovese, Corinto Nero and Gamay derived from self-pollination and emasculation with inflorescence bagging. **Figure S12.** Clusters obtained from Corinto Nero inflorescences after emasculation and manual pollination with Nebbiolo pollen.

## Data Availability

All data generated during this study are included within the article and in its supplementary information files or are available from the corresponding author on reasonable request. BioSample metadata can be found in the BioSample database (http://www.ncbi.nlm.nih.gov/biosample/) under accession numbers SAMN17282791-SAMN17282807. The partial nucleotide sequences of VIT_02s0025g03330, VIT_06s0004g03800, VIT_11s0016g03590, VIT_11s0016g05820 and VIT_14s0083g00910 are available in the GenBank data libraries (https://www.ncbi.nlm.nih.gov/genbank/) under accession numbers MW464209-MW464218.

## Abbreviations

AHA: Autoinhibited H^+^ ATPase
Arg: Arginine
CAPS: Cleaved Amplified Polymorphic Sequence
CCR4-NOT: Carbon Catabolite Repression-Negative On TATA-less
DNA: DeoxyriboNucleic Acid
E-L: Eichhorn-Lorenz
ELISA: Enzyme-Linked ImmunoSorbent Assay
FDR: First Division Restitution
FEM: Fondazione Edmund Mach
INDEL: INsertion-DELetion
INO80: INOsitol-requiring 80
IPSP: Institute for Sustainable Plant Protection
Leu: Leucine
LG: Linkage Group
Met: Methionine
miRNA: microRNA
mRNA: messenger RNA
OP: open
PCR: Polymerase Chain Reaction
QTL: Quantitative Trait Locus
RNA: RiboNucleic Acid
RNA-Seq: RNA-Sequencing
SDI: Seed Development Inhibitor
SDR: Second Division Restitution
SNP: Single-Nucleotide Polymorphism
SP: Self
SSR: Simple Sequence Repeat
SWR1: SWi2/snf2-Related 1
Thr: Threonine
TTC: 2,3,5-Triphenyl Tetrazolium Chloride
VIVC: *Vitis* International Variety Catalogue
VvAGL11: *Vitis vinifera* AGAMOUS-LIKE 11
