# Supplementary material for: Somatic variants for seed and fruit set in grapevine
Source: BMC Plant Biol. 2021 Mar 13;21:135. doi: 10.1186/s12870-021-02865-2 (PMC7955655; doi:10.1186/s12870-021-02865-2)
Supplement: Supplementary file 2 — Additional file 2: Figure S1. Trait stability in multiple seasons and locations upon open-pollination. [file 12870_2021_2865_MOESM2_ESM.pdf]

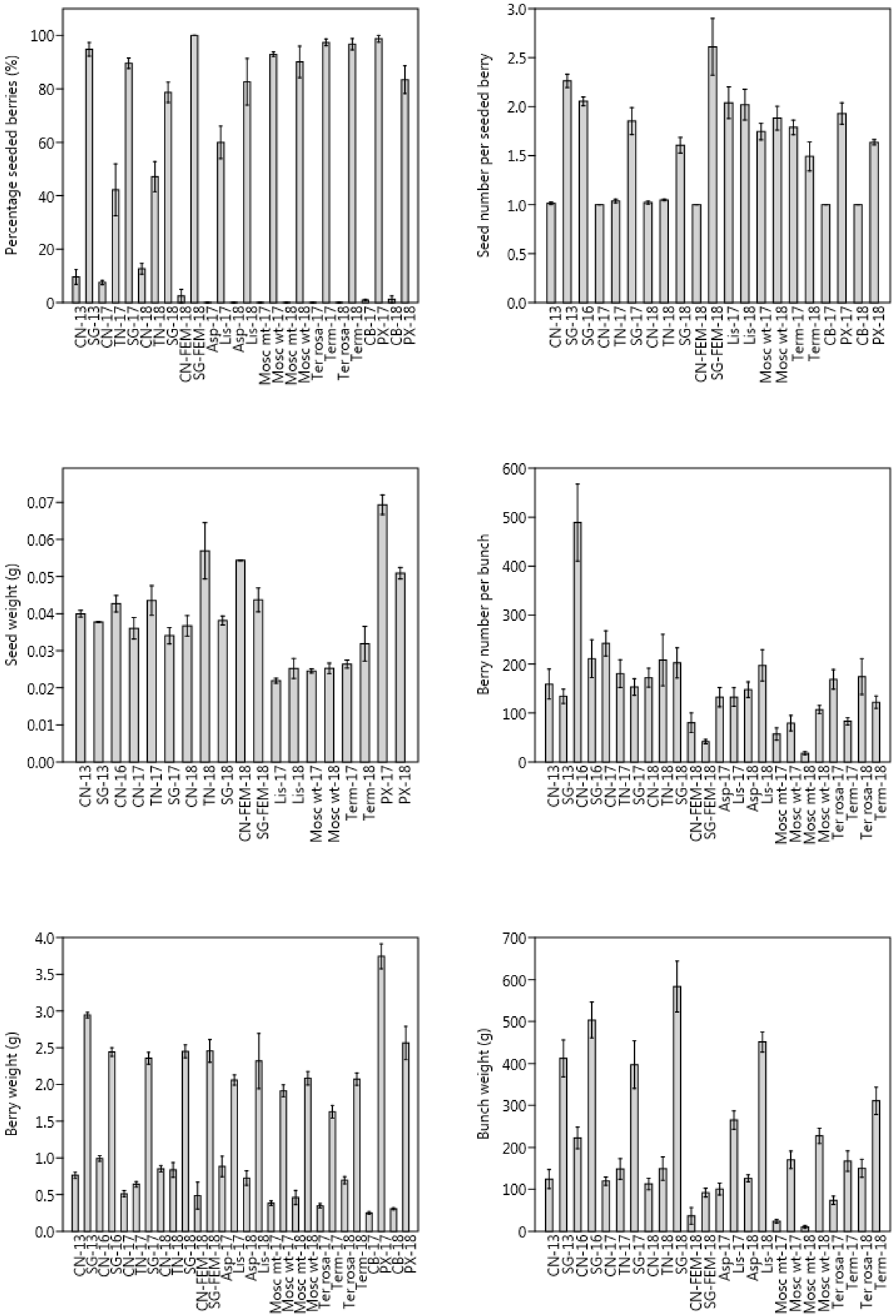

Percentage seeded berries (%)
CN-13 SG-13 CN-17 TN-17 SG-17 CN-18 TN-18 SG-18 CN-FEM-18 SG-FEM-18 Asp-17 Lis-17 Asp-18 Lis-18 Mosc mt-17 Mosc wt-17 Mosc mt-18 Mosc wt-18 Ter rosa-17 Term-17 Ter rosa-18 Term-18 CB-17 PX-17 CB-18 PX-18
Seed number per seeded berry
CN-13 SG-13 SG-16 CN-17 TN-17 SG-17 CN-18 TN-18 SG-18 CN-FEM-18 SG-FEM-18 Lis-17 Lis-18 Mosc wt-17 Mosc wt-18 Term-17 Term-18 CB-17 PX-17 CB-18 PX-18
Seed weight (g)
CN-13 SG-13 CN-16 CN-17 TN-17 SG-17 CN-18 TN-18 SG-18 CN-FEM-18 SG-FEM-18 Lis-17 Lis-18 Mosc wt-17 Mosc wt-18 Term-17 Term-18 PX-17 PX-18
Berry number per bunch
CN-13 SG-13 CN-16 SG-16 CN-17 TN-17 SG-17 CN-18 TN-18 SG-18 CN-FEM-18 SG-FEM-18 Asp-17 Lis-17 Asp-18 Lis-18 Mosc mt-17 Mosc wt-17 Mosc mt-18 Mosc wt-18 Ter rosa-17 Term-17 Ter rosa-18 Term-18
Berry weight (g)
CN-13 SG-13 CN-16 SG-16 CN-17 TN-17 SG-17 CN-18 TN-18 SG-18 CN-FEM-18 SG-FEM-18 Asp-17 Lis-17 Asp-18 Lis-18 Mosc mt-17 Mosc wt-17 Mosc mt-18 Mosc wt-18 Ter rosa-17 Term-17 Ter rosa-18 Term-18 CB-17 PX-17 CB-18 PX-18
Bunch weight (g)
CN-13 SG-13 CN-16 SG-16 CN-17 TN-17 SG-17 CN-18 TN-18 SG-18 CN-FEM-18 SG-FEM-18 Asp-17 Lis-17 Asp-18 Lis-18 Mosc mt-17 Mosc wt-17 Mosc mt-18 Mosc wt-18 Ter rosa-17 Term-17 Ter rosa-18 Term-18

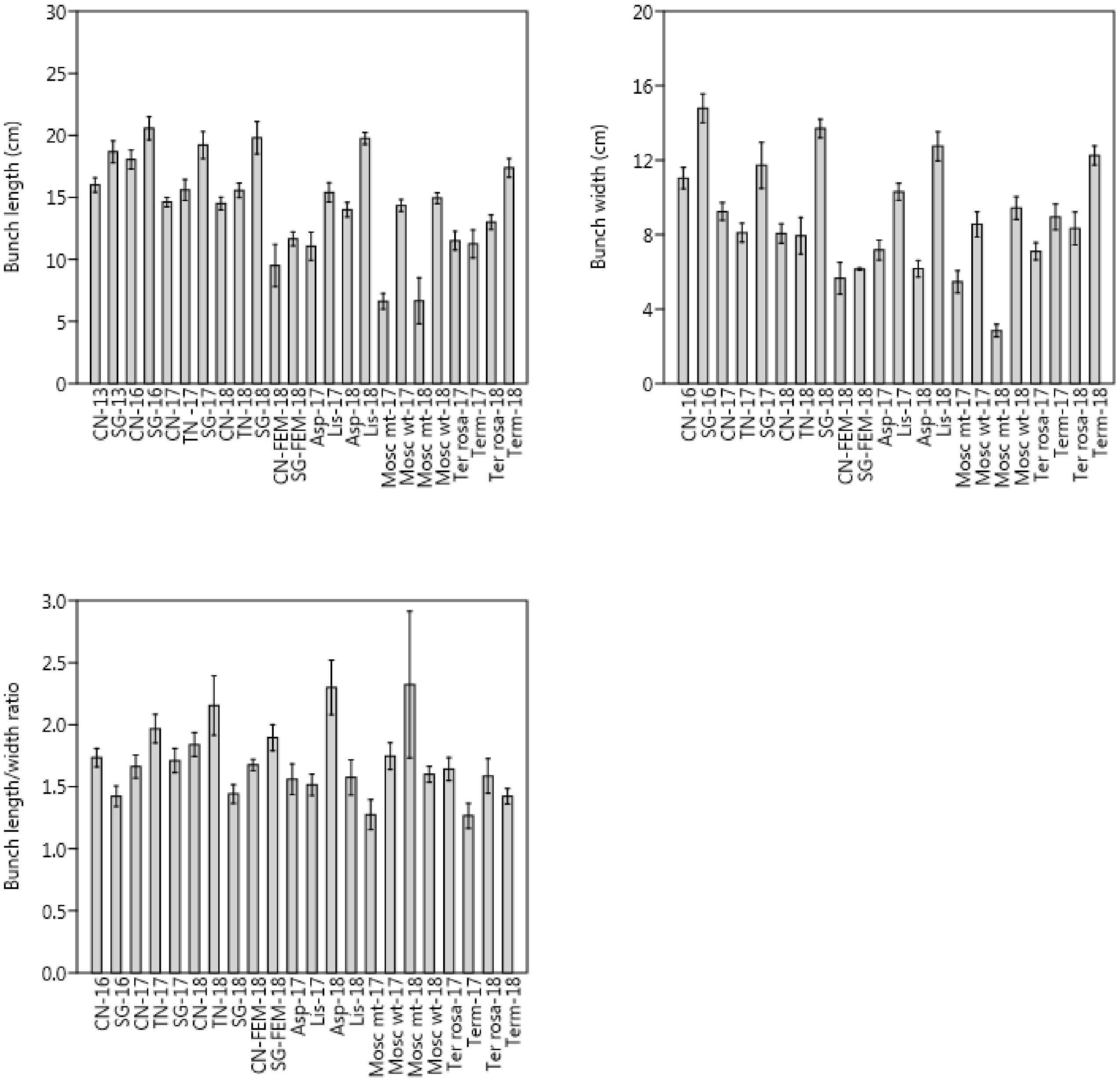

Figure S1: Trait stability in variant pairs that were phenotyped in multiple seasons (2013, 2016, 2017, 2018) and locations (IPSP and FEM) upon open-pollination. For each accession a mean value was calculated from distinct bunches. Bars correspond to standard errors. Abbreviations: CN = Corinto Nero, TN = Termarina Nera, SG = Sangiovese, Asp = Aspirant-false, Lis = Liseiret, Mosc mt = Moscato Bianco mutant, Mosc wt = Moscato Bianco, Ter rosa = Termarina Rosa, Term = Termarone, CB = Corinto Bianco, PX = Pedro Ximenez.
