# Supplementary material for: Somatic variants for seed and fruit set in grapevine
Source: BMC Plant Biol. 2021 Mar 13;21:135. doi: 10.1186/s12870-021-02865-2 (PMC7955655; doi:10.1186/s12870-021-02865-2)
Supplement: Supplementary file 3 — Additional file 3: Figure S2. Relationship between berry size and presence of normal seeds. [file 12870_2021_2865_MOESM3_ESM.pdf]

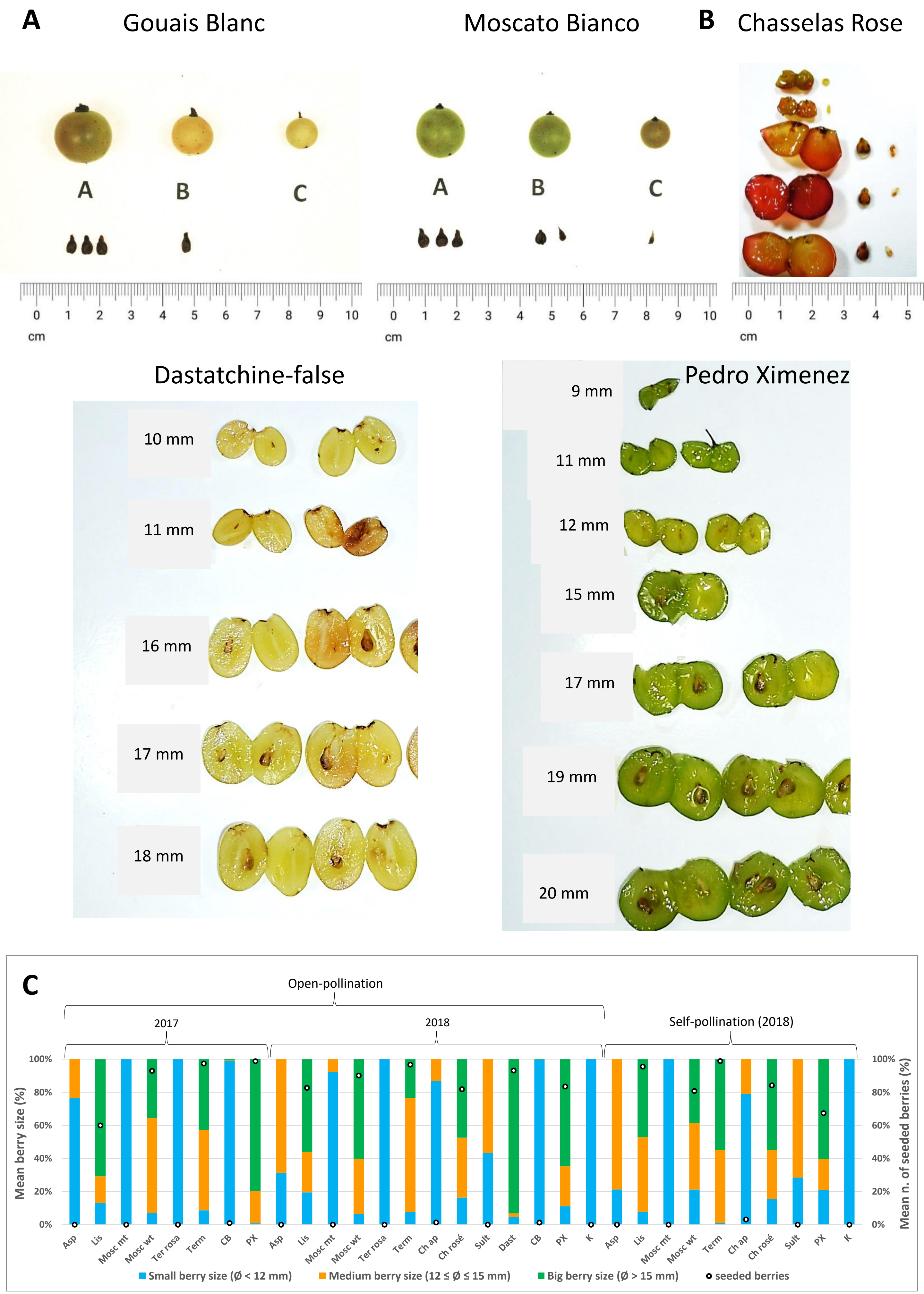

A
Gouais Blanc
Moscato Bianco
B
Chasselas Rose
A
B
C
A
B
C
0 1 2 3 4 5 6 7 8 9 10
cm
0 1 2 3 4 5 6 7 8 9 10
cm
0 1 2 3 4 5
cm
Dastatchine-false
10 mm
11 mm
16 mm
17 mm
18 mm
Pedro Ximenez
9 mm
11 mm
12 mm
15 mm
17 mm
19 mm
20 mm
C
Open-pollination
2017
2018
Self-pollination (2018)
Mean berry size (%)
Mean n. of seeded berries (%)
100%
80%
60%
40%
20%
0%
Asp Lis Mosc mt Mosc wt Ter rosa Term CB PX
Asp Lis Mosc mt Mosc wt Ter rosa Term Ch ap Ch rosé Sult Dast CB PX K
Asp Lis Mosc mt Mosc wt Term Ch ap Ch rosé Sult PX K
Small berry size (Ø < 12 mm)
Medium berry size (12 ≤ Ø ≤ 15 mm)
Big berry size (Ø > 15 mm)
seeded berries

Figure S2: Relationship between berry size and presence of normal seeds. (A) Classification of berries according to size in Gouais Blanc and in Moscato Bianco at IPSP; the prevalent type of seeds or seed traces is shown below. (B) Seeds from berries of different size (as assessed with an *ad hoc* aluminum sizer card) in the FEM accessions Chasselas Rose, Dastatchine-false and Pedro Ximenez. (C) Percentage distribution of berries according to size and content of fully developed seeds. The percentage of small, medium and large berries was calculated from the total number of berries per bunch, while the percentage of seeded berries was established on the total number of berries opened for seed examination (it was a representative portion of the total number of berries when this number was too big). For each combination of accession, season and pollination treatment, from one to ten clusters were analyzed and an average value was calculated. Abbreviations: Asp = Aspirant-false, Lis = Liseiret, Mosc mt = Moscato Bianco mutant, Mosc wt = Moscato Bianco, Ter rosa = Termarina Rosa, Term = Termarone, Ch ap = Chasselas apyrène, Ch rose = Chasselas Rose, Sult = Sultanina, Dast = Dastatchine-false, CB = Corinto Bianco, PX = Pedro Ximenez, K = Corinthe Noir.
