# Supplementary material for: Somatic variants for seed and fruit set in grapevine
Source: BMC Plant Biol. 2021 Mar 13;21:135. doi: 10.1186/s12870-021-02865-2 (PMC7955655; doi:10.1186/s12870-021-02865-2)
Supplement: Supplementary file 4 — Additional file 4: Figure S3. Percentage distribution of Sangiovese and Corinto Nero berries according to seed content in two pollination conditions. [file 12870_2021_2865_MOESM4_ESM.pdf]

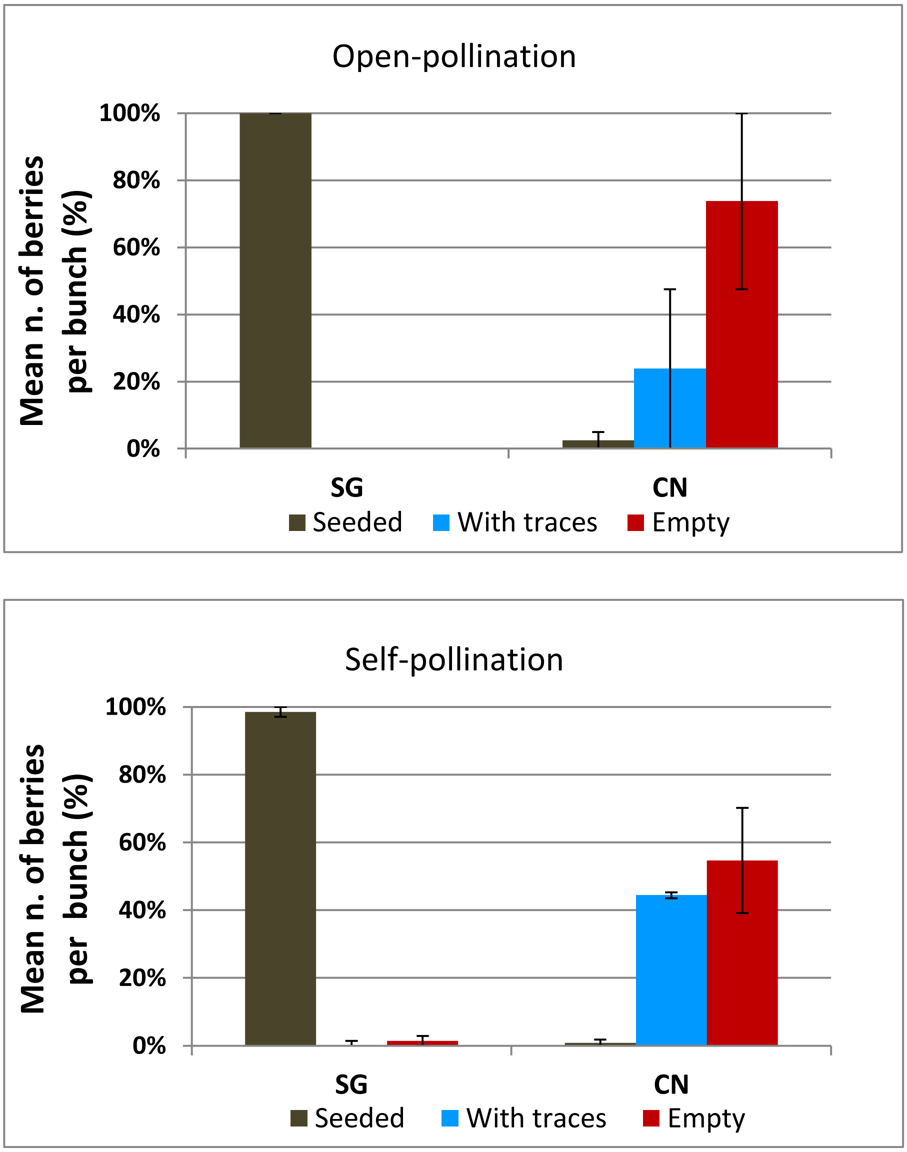

Figure S3: Percentage distribution of Sangiovese (SG) and Corinto Nero (CN) berries according to seed content in two pollination conditions. Berries were considered as seeded when they accommodated at least one apparently normal seed (and possibly also traces); seedless berries with traces contained only traces (attributed to ovules); seedless empty berries were apparently devoid of any rudiment. Mean values were calculated from 2 or 3 bunches (derived from open- and self-pollination, respectively) that were collected in FEM in 2018. Bars correspond to standard errors.
