# Supplementary material for: Somatic variants for seed and fruit set in grapevine
Source: BMC Plant Biol. 2021 Mar 13;21:135. doi: 10.1186/s12870-021-02865-2 (PMC7955655; doi:10.1186/s12870-021-02865-2)
Supplement: Supplementary file 5 — Additional file 5: Inspection of traces and seeds extracted from berries at veraison for the seedless accessions Aspirant (Figure S4), Chasselas apyrène (Figure S5), Corinto Nero (Figure S6), Termarina Rosa and Moscato Bianco mutant (Figure S7), Corinthe Noir and Sultanina (Figure S8), and for the seeded cultivars Liseiret, Moscato Bianco, Termarone and Sangiovese (Figure S9). [file 12870_2021_2865_MOESM5_ESM.pdf]

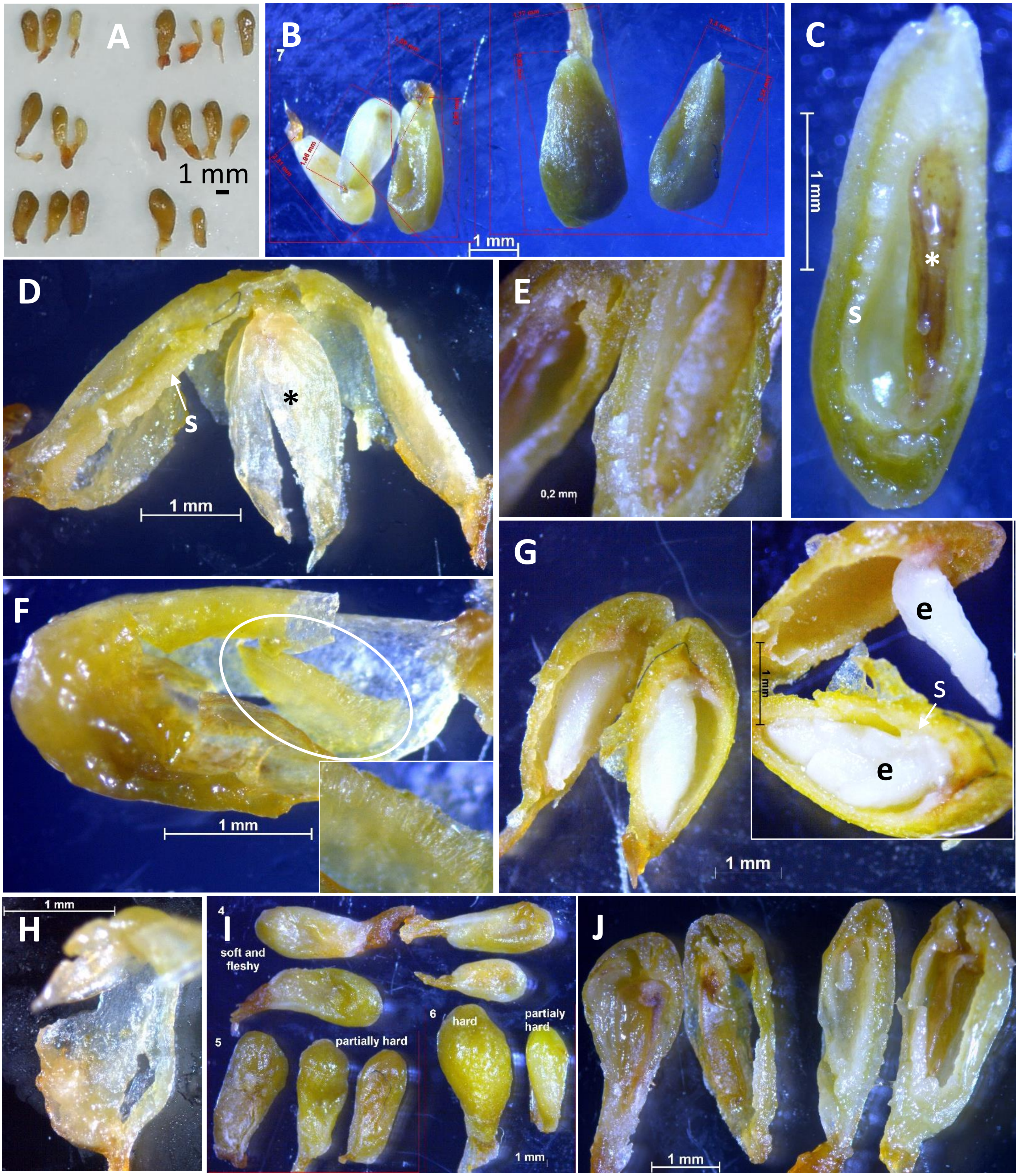

Figure S4: Traces extracted from ten smaller (A-C) and ten bigger (D-J) berries of Aspirant collected at veraison. Most of the traces from the smaller berries were soft (A-B), although some of the biggest ones were more differentiated presenting a partially developed sclerenchyma and, in some cases, a degenerated tissue that could correspond to a degenerated nucellus or endosperm (C). In general, traces extracted from the biggest berries were more developed, some of them were totally soft, others were partially hard, usually in the chalazal end, and a few of them, the biggest ones, were almost completely hard (I). After dissection, different seed structures could be appreciated in most of these traces, such as a partially developed

sclerenchyma, usually in the chalazal end (D-G, J) and what could be a degenerated nucellus or endosperm (D). One of the traces presented a partially developed endosperm (G). The smallest traces were soft and no structures could be recognized (H). No rumination ingrowths were observed. e: endosperm, s: sclerenchyma, (*): degenerated tissue, probably degenerated nucellus or endosperm.

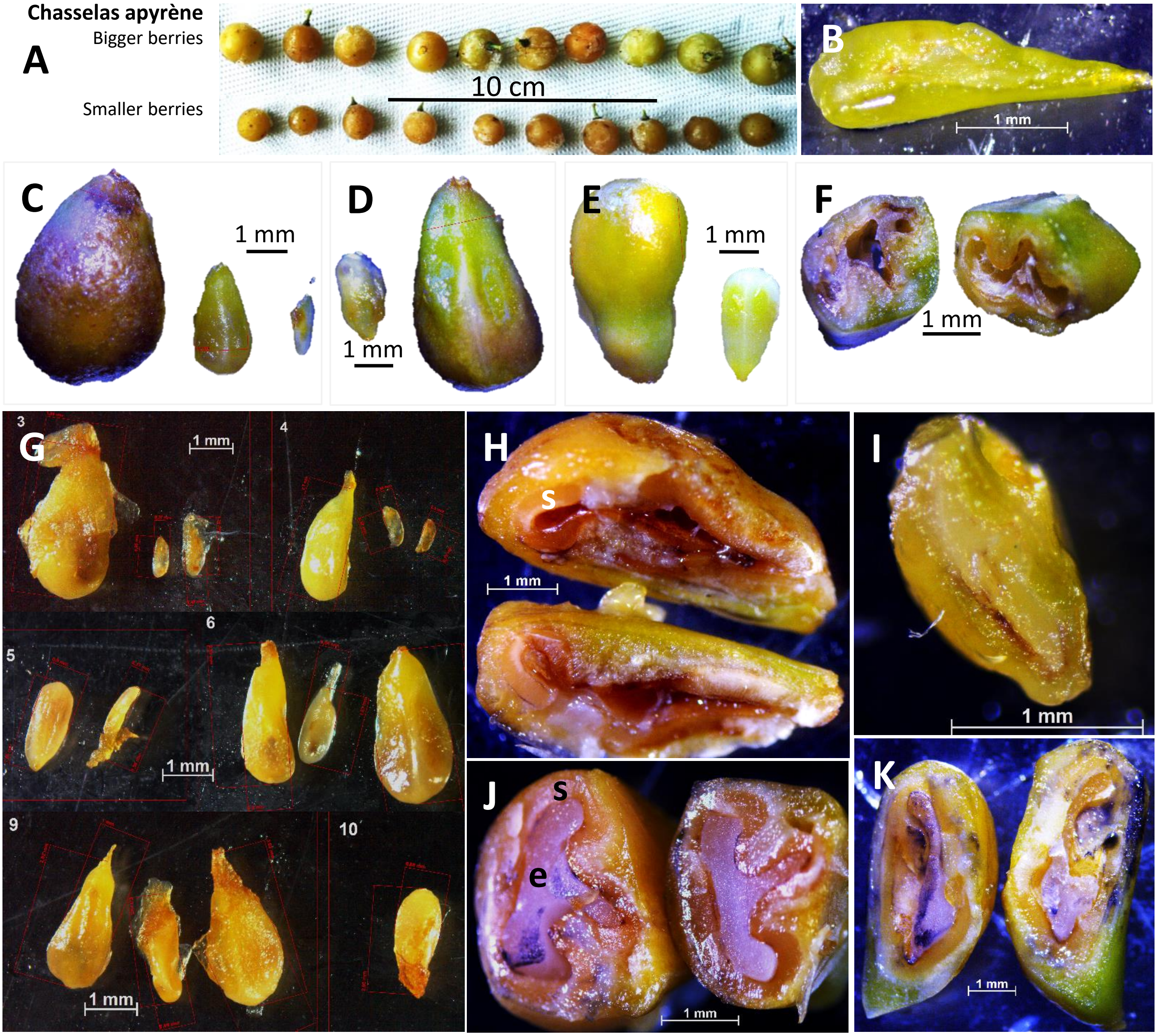

Figure S5: Chasselas apyrène berries collected at veraison for inspection of traces and, eventually, seeds (A). All traces extracted from smaller berries were soft, heterogeneous in size and no structures were identified after dissection (B, G). Bigger berries also contained soft traces together with bigger and more developed seed traces and/or seeds (C-E). Some of these seed traces were in an advanced stage of development with a well developed seed coat, sclerenchyma, complete endosperm rumination (F, H, J-K); however, some of them lacked an endosperm (F, H). Others, instead, were in earlier stages of development, the different structures could not be distinguished and rumination had not taken place (I). Black spots in the endosperm in some pictures (J-K) are due to the cut with the scalpel, they are not intrinsic of the tissue. e: endosperm, s: sclerenchyma.

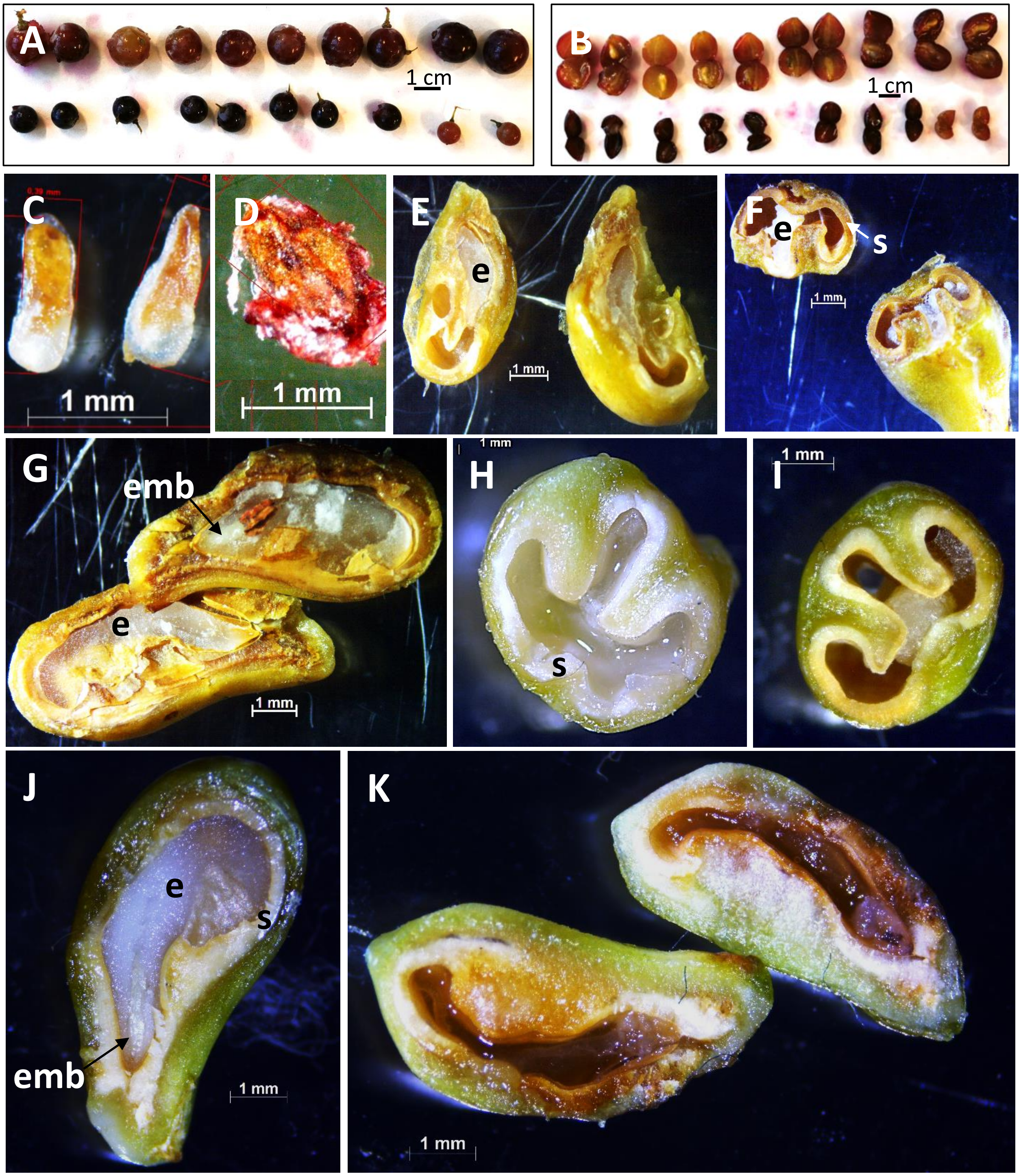

Figure S6. Corinto Nero berries collected at veraison for inspection of traces and, eventually, seeds. Close and opened berries (A-B); bigger berries in the upper part and smaller berries in the lower part. Smaller berries contained just very small and soft traces, impossible to dissect without destroying them because of the reduced size (C-D). Bigger berries usually accommodated one seed (E-K). Despite that all seeds sank in water, when dissected, some of them presented a partially developed endosperm with cavities and no embryo, as shown in longitudinal (E) and transversal sections (F, I). In others the endosperm had a liquid or gel-like consistency, as shown in transversal (H) and longitudinal (K) sections. Other seeds, instead, were completely developed and longitudinal sections evidenced the presence of an embryo (G, J). e: endosperm, emb: embryo, s: sclerenchyma.

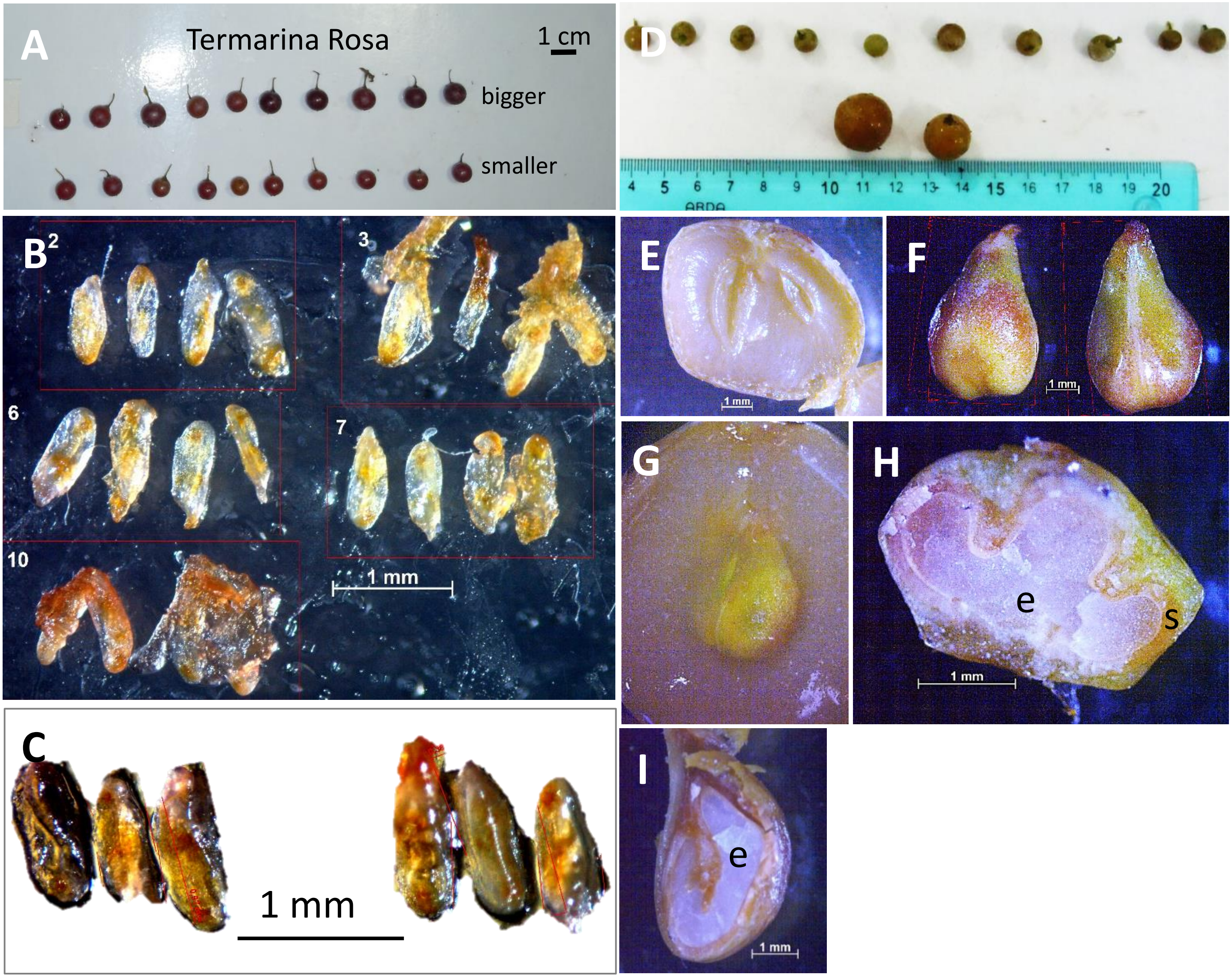

Figure S7: Termarina Rosa (A-C) and Moscato Bianco mutant (D-I) berries at veraison with traces and seeds extracted for analysis. Differences in berry size were not so evident in Termarina Rosa (A). Traces extracted from smaller (B) and bigger (C) berries were similar: very small and soft. It was not possible to dissect them because they were destroyed due to their reduced size and fragility. Among all the collected berries of Moscato Bianco mutant only two bigger berries (the size difference was clear) were found (D). All smaller inspected berries did not contain traces and in some of them signs of the locules which harbored the ovules could be seen (E). Both bigger berries contained two seeds (F-G). Transversal and longitudinal sections of the seeds were performed and the structures of a normal seed were observed (H-I). e: endosperm, s: sclerenchyma.

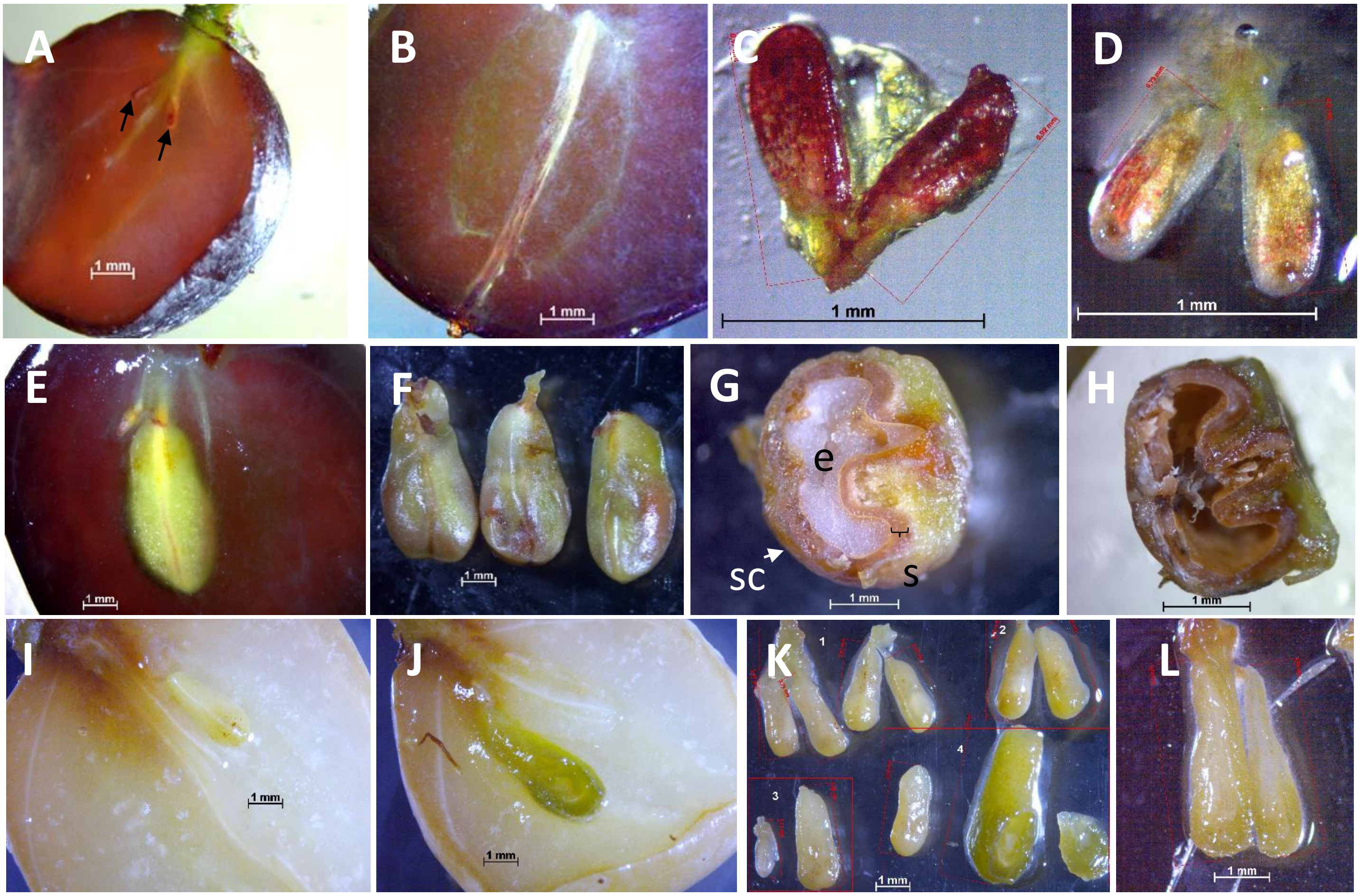

Figure S8: Small (A-B) and large (E) berries of Corinthe Noir and berries of Sultanina (I-J) inspected at veraison with the extracted traces and seeds (C-D, F-H, K-L). Most of the smaller berries of Corinthe Noir accommodated traces (A) while others had no traces at all (B). These traces were very small and soft (C-D), it was impossible to dissect them because they were destroyed due to their reduced size and fragility. Bigger berries of Corinthe Noir contained a seed and sometimes also traces (E). Ventral side of three seeds extracted from large berries (F). Transversal section of two Corinthe Noir seeds: one showed what seems a normal endosperm (G) and the other was empty (H). The analyzed berries of Sultanina were homogeneous in size and presented seed traces (I-J). The examined traces were heterogeneous in size, all were soft (even the biggest ones) (K). Dissection was only successful for a few of them because they got damaged due to their size and soft consistency. Anyway, no defined structure could be observed in the few successful longitudinal sections (L). e: endosperm, s: sclerenchyma, black arrows indicate the small traces within the Corinthe Noir berry.

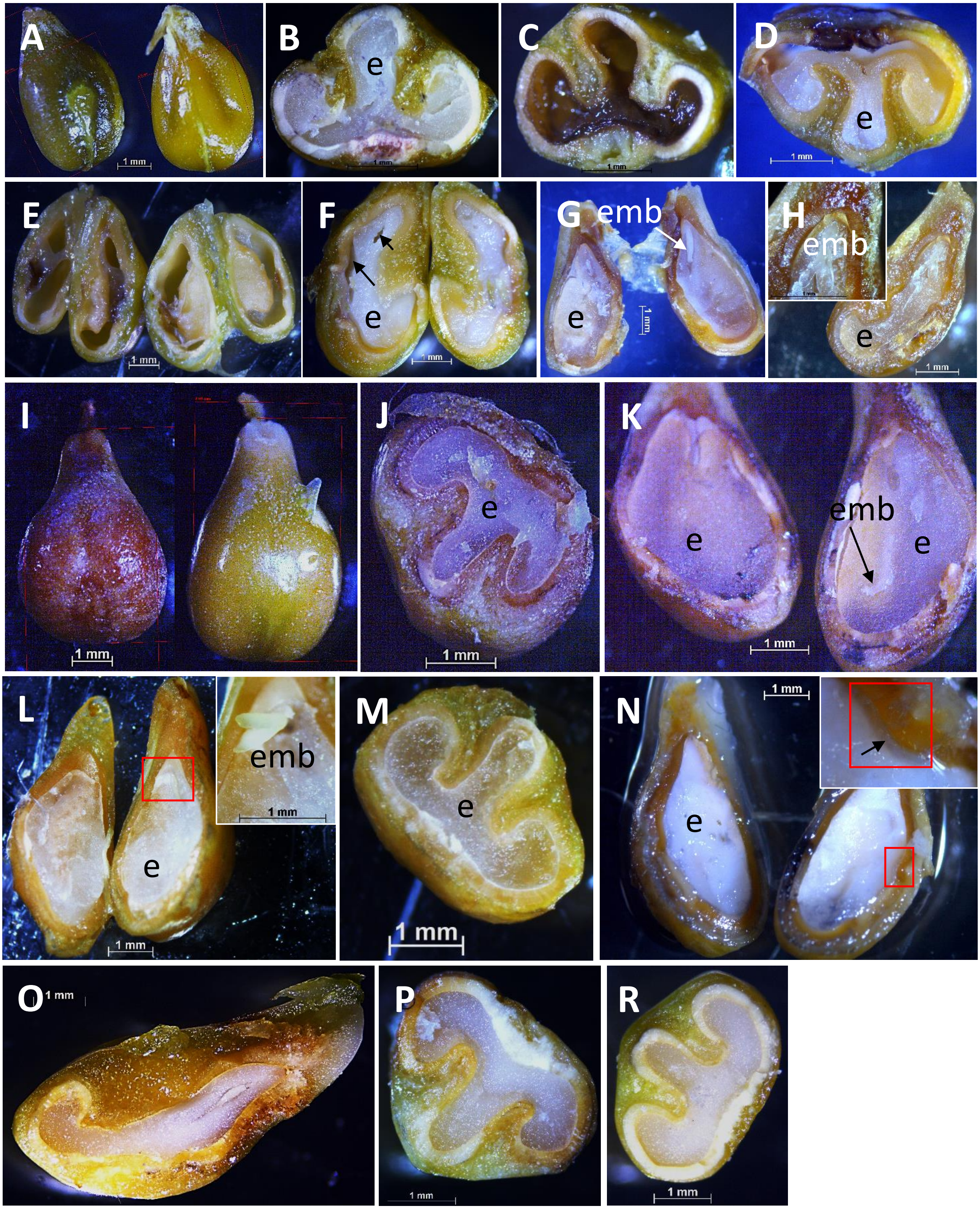

Figure S9: Seeds extracted from the berries of the seeded cultivars Liseiret (A-H), Moscato Bianco (I-K), Termarone (L-N) and Sangiovese (O-R) collected at veraison. Most of Liseiret extracted seeds (67%) were potentially vital according to the floatation test, presenting a well-developed endosperm and an embryo, as shown by transversal (B) and longitudinal sections (G-H). Seeds that floated were empty (E), the endosperm was degenerated (C), in some cases the endosperm had a normal appearance, but it seemed that its development was arrested (D) or it looked like a normal endosperm but cavities (black arrows in F) within it or between the endosperm and the integument could be observed, which might indicate initiation of

endosperm degradation (D, F). All extracted seeds of Moscato Bianco and Sangiovese were potentially vital containing a well-developed endosperm and an embryo could be seen in most of them (J-K, O-R). This was also observed for almost all extracted seeds of Termarone, for which only one seed floated in water. When dissected, it contained an apparently well-developed endosperm, however very small spaces (black arrow) between it and the integument could be appreciated (N). e: endosperm, emb: embryo.
