# Supplementary material for: Somatic variants for seed and fruit set in grapevine
Source: BMC Plant Biol. 2021 Mar 13;21:135. doi: 10.1186/s12870-021-02865-2 (PMC7955655; doi:10.1186/s12870-021-02865-2)
Supplement: Supplementary file 6 — Additional file 6: Figure S10. Sangiovese and Corinto Nero pistils at six phenological stages, with details of ovules/seed traces for which length and width were measured. [file 12870_2021_2865_MOESM6_ESM.pdf]

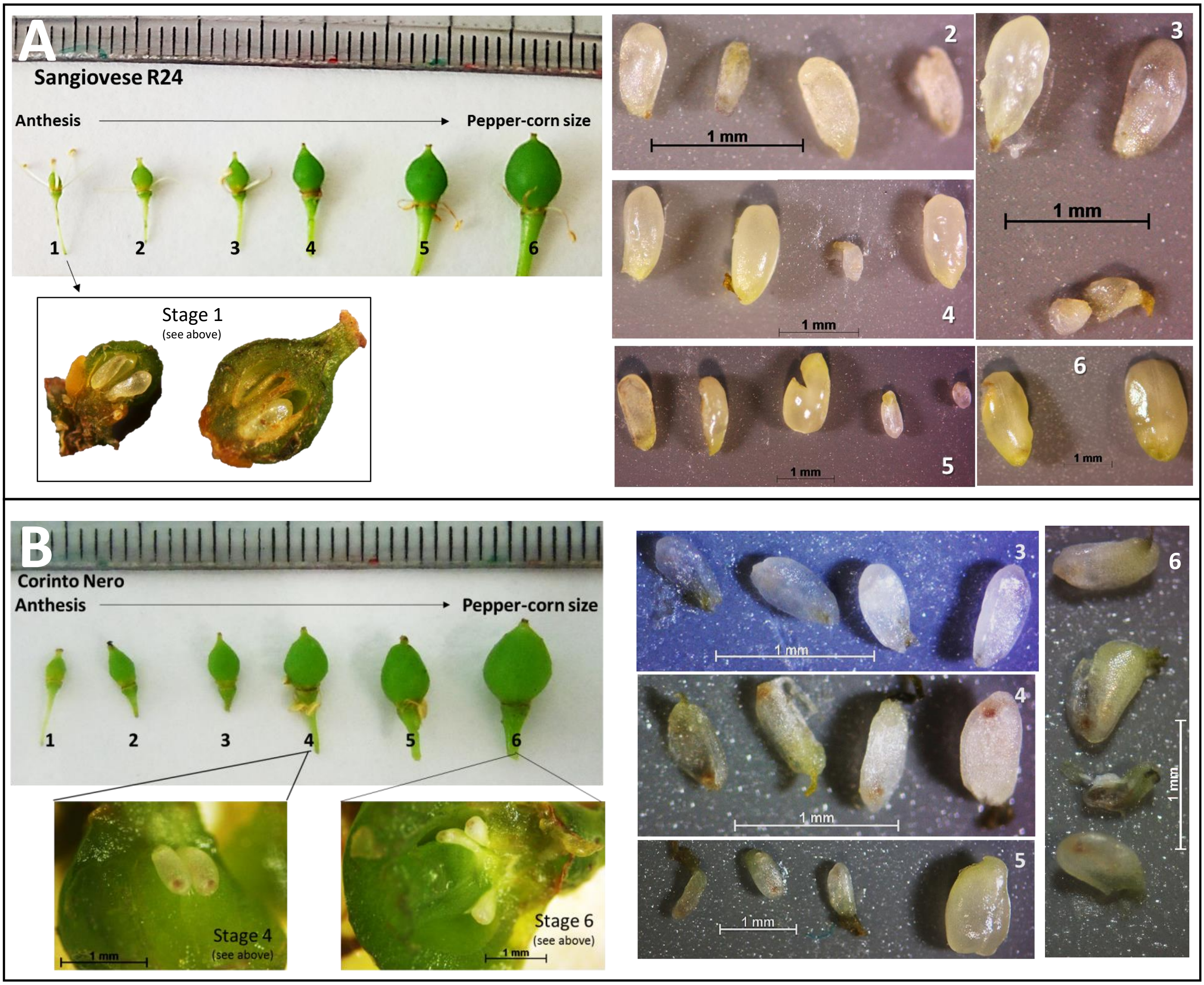

Figure S10: On the left, pistils selected for dissection at six phenological stages from flowering (stage 1) to berries pepper-corn size (stage 6) and some longitudinal sections showing ovules/seed traces in Sangiovese (A) and its seedless variant, Corinto Nero (B); on the right, detailed pictures of the ovules/seed traces for which length and width were measured (the number in the upper or lower right corner of the pictures indicates the stage of the pistil from which they were extracted).
