# Supplementary material for: Somatic variants for seed and fruit set in grapevine
Source: BMC Plant Biol. 2021 Mar 13;21:135. doi: 10.1186/s12870-021-02865-2 (PMC7955655; doi:10.1186/s12870-021-02865-2)
Supplement: Supplementary file 7 — Additional file 7: Figure S11. Clusters of Sangiovese, Corinto Nero and Gamay derived from self-pollination and emasculation with inflorescence bagging. Figure S12. Clusters obtained from Corinto Nero inflorescences after emasculation and manual pollination with Nebbiolo pollen. [file 12870_2021_2865_MOESM7_ESM.pdf]

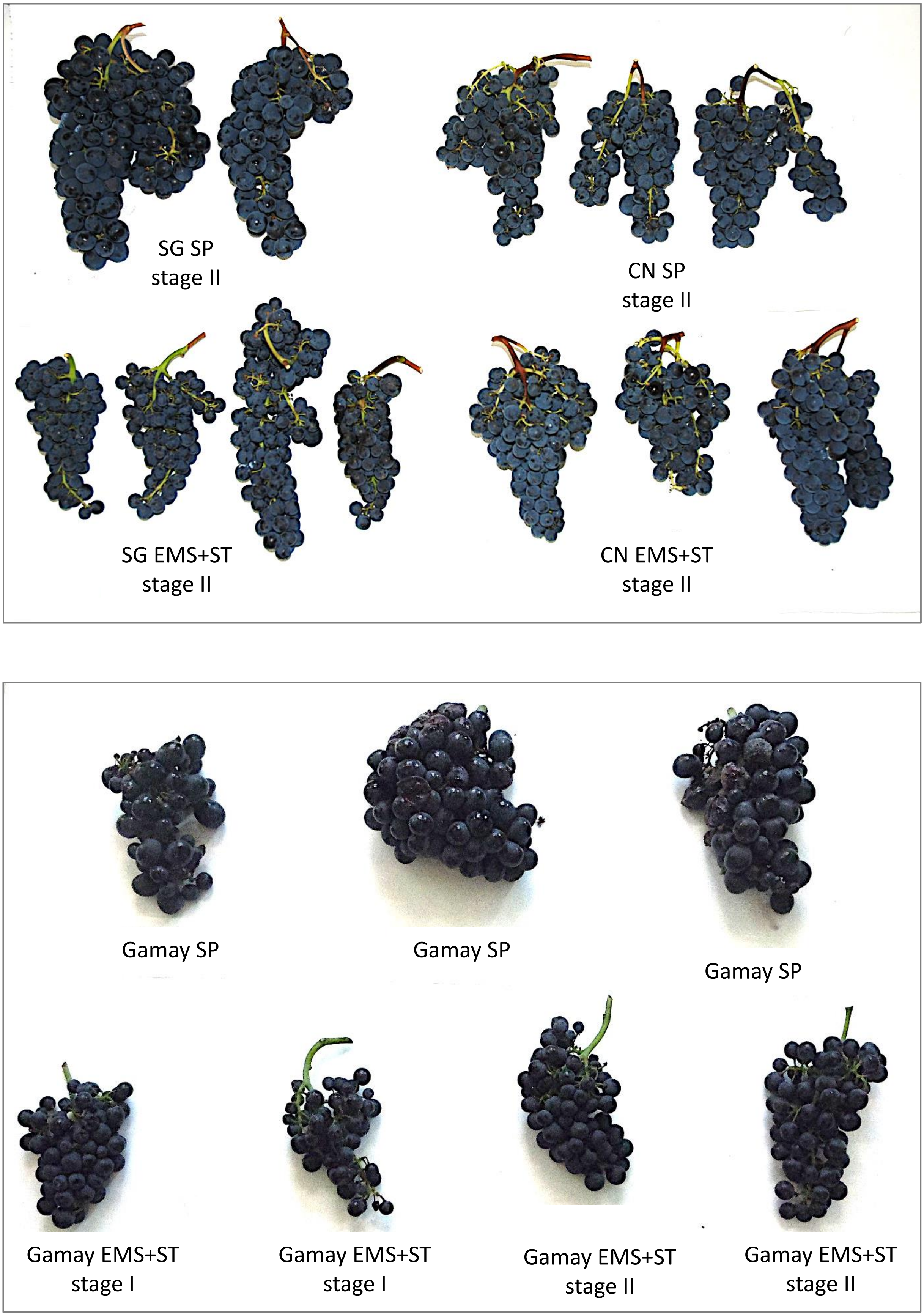

Figure S11: Clusters of Sangiovese, Corinto Nero and Gamay derived from self-pollination (control) and emasculation with inflorescence bagging. Abbreviations: SG = Sangiovese, CN = Corinto Nero, SP = self-pollinated, EMS+ST = emasculated (without stigma removal), stage I = stage E-L 15, stage II = stage E-L 18 of the modified Eichhorn-Lorenz scheme (Coombe, 1995).

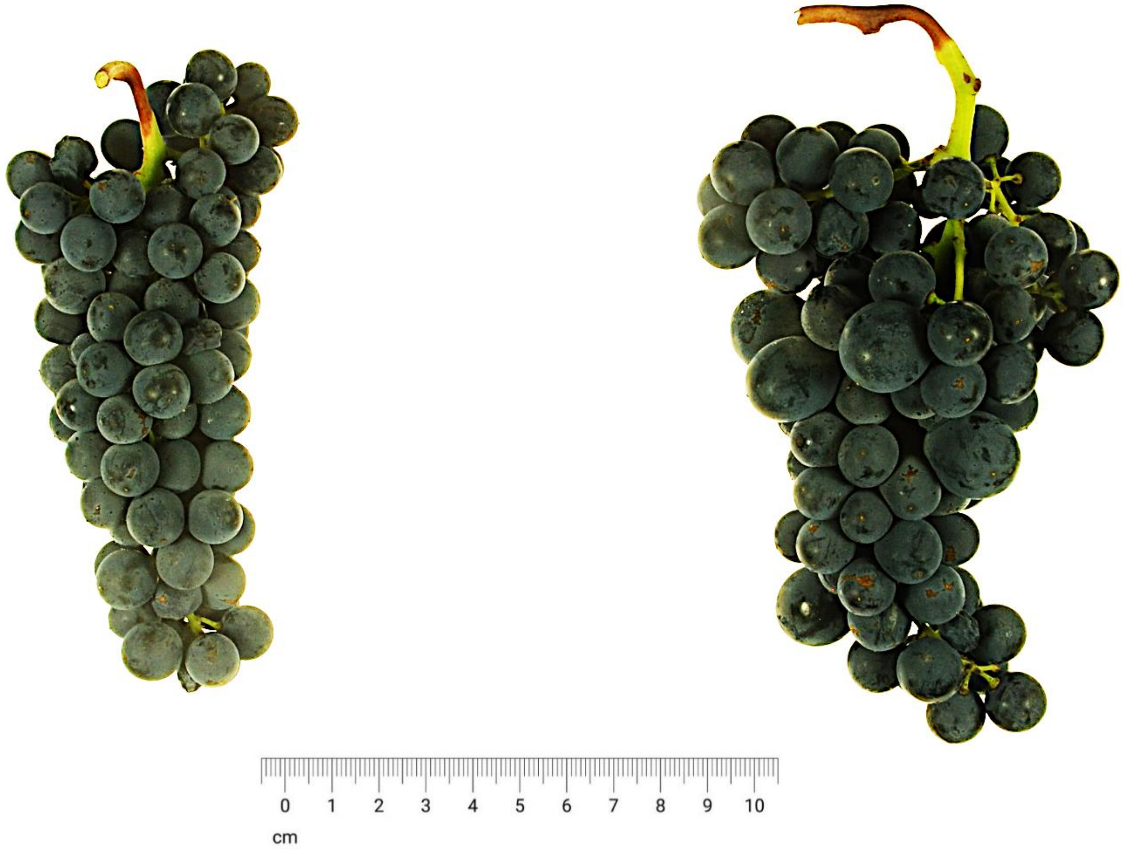

Figure S12: An example of clusters obtained from Corinto Nero inflorescences after emasculation and manual pollination with Nebbiolo pollen in 2013.
